# Roots and Panicles of the C4 Model Grasses *Setaria viridis* (L). and *S. pumila* Host Distinct Bacterial Assemblages With Core Taxa Conserved Across Host Genotypes and Sampling Sites

**DOI:** 10.3389/fmicb.2018.02708

**Published:** 2018-11-12

**Authors:** Carolina Escobar Rodríguez, Birgit Mitter, Livio Antonielli, Friederike Trognitz, Stéphane Compant, Angela Sessitsch

**Affiliations:** Bioresources Unit, Center for Health & Bioresources, AIT Austrian Institute of Technology GmbH, Vienna, Austria

**Keywords:** *Setaria* microbiota, model grass, core microbiota, root endophytes, inflorescence endophytes, bacterial community drivers, bacterial microbiota

## Abstract

Virtually all studied plant tissues are internally inhabited by endophytes. Due to their relevance for plant growth and health, bacterial microbiota of crop plants have been broadly studied. In plant microbiome research the root is the most frequently addressed environment, whereas the ecology of microbiota associated with reproductive organs still demands investigation. In this work, we chose the model grasses *Setaria viridis* and *Setaria pumila* to better understand the drivers shaping bacterial communities associated with panicles (representing a reproductive organ) as compared to those associated with roots. We collected wild individuals of both grass species from 20 different locations across Austria and investigated the bacterial assemblages within roots and ripe grain-harboring panicles by 16S rRNA gene-based Illumina sequencing. Furthermore, plant samples were subjected to genotyping by genetic diversity-focused Genotyping by Sequencing. Overall, roots hosted more diverse microbiota than panicles. Both the plant organ and sampling site significantly shaped the root and panicle-associated microbiota, whereas the host genotype only affected root communities. In terms of community structure, root-specific assemblages were highly diverse and consisted of conserved bacterial taxa. In contrast, panicle-specific communities were governed by *Gammaproteobacteria*, were less diverse and highly origin-dependent. Among OTUs found in both plant tissues, relative abundances of *Gammaproteobacteria* were higher in panicles, whereas *Rhizobiales* dominated root communities. We further identified core and non-core taxa within samples of both *Setaria* species. Non-core taxa included members of the *Saccharibacteria* and *Legionelalles*, while core communities encompassed eleven OTUs of seven bacterial orders, together with a set of ten panicle-enriched OTUs. These communities were widespread across root and panicle samples from all locations, hinting toward an evolved form of mutualism through potential vertical transmission of these taxa within *Setaria* species.

## Introduction

Plants host highly diverse microbial communities, which interact with their host in multiple ways ranging from mutualism to commensalism or pathogenicity (Newton et al., 2010; Hardoim et al., 2015). Rhizosphere microbiota are diverse and largely influenced by plant roots and exudates (Mendes et al., 2013), whereas endosphere microbiota are generally less diverse and comprise microorganisms, which spend at least part of their life cycle inside plants (Hardoim et al., 2015). Most plant tissues are colonized by endophytes, either bacteria and/or fungi, however, they host distinct microbial assemblages (Compant et al., 2010, 2011; Coleman-Derr et al., 2016). The soil, which represents an extremely diverse ecosystem, is considered as the main source of plant microbiota. Numerous studies have shown that the soil environment is a key driver of root-associated microbial assemblages, both in the rhizosphere (Rasche et al., 2006a; Peiffer et al., 2013; Edwards et al., 2015; Pfeiffer et al., 2017) as well as in the root endosphere (Gottel et al., 2011; Edwards et al., 2015). The soil environment was also shown to influence microbiota of above-ground tissues such as stems (Rasche et al., 2006b), fruits (Zarraonaindia et al., 2015) or seeds (Klaedtke et al., 2015).

Whereas rhizosphere and root bacterial microbiota have been frequently analyzed, less investigated are those associated with reproductive organs such as flowers (Shade et al., 2013), or disseminative organs such as fruits (Compant et al., 2011; Glassner et al., 2017) or seeds (Johnston-Monje and Raizada, 2011; Barret et al., 2015; Klaedtke et al., 2015). Generally, roots are colonized by a subset of rhizosphere bacteria, which are able to enter roots and reach the cortical cell layer or endodermis. From there, some bacterial endophytes migrate via the xylem or intracellular spaces to above ground tissues (Hardoim et al., 2015), however, vertical spread of bacteria is slow and may take several weeks (Compant et al., 2008).

Reproductive and disseminative organs carry microorganisms, which are potentially vertically transmitted to progeny plants, and therefore may represent an important source of microorganisms involved in early plant growth development. Specific fungal endophytes such as *Epichloë* species (*Neotyphodium* for anamorphs) are known to be vertically transmitted via seeds (Saikkonen et al., 2002; Leuchtmann et al., 2014), whereas for bacterial endophytes this has been rarely indicated (Johnston-Monje and Raizada, 2011). Johnston-Monje and Raizada (2011) found bacterial groups conserved across different *Zea* genotypes irrespective of differences in genotype, degree of domestication or geographic origin and concluded that these might have been constantly vertically transmitted.

It is well known that bacterial endophytes may migrate to above-ground tissues, which include reproductive and disseminative organs (Aleklett et al., 2014; Truyens et al., 2015; Glassner et al., 2017). Nevertheless, origin and ecology of these microbiota are poorly understood, especially in wild relatives of agronomic crops at their natural habitats (Aleklett et al., 2014; Berg and Raaijmakers, 2018). Hence the aim of this study is to advance our understanding on the ecology of bacterial communities of disseminative organs. As a model plant we used *Setaria* spp., i.e., *Setaria pumila* and *Setaria* *viridis*, both are ubiquitous weed species. The latter is the weedy relative of the cereal crop *Setaria italica* (foxtail millet) and has been suggested as a model species for crop plants of the Andropogoneae tribe such as maize, sorghum, *Miscanthus*, and sugarcane (Li and Brutnell, 2011). Studies using *Setaria* species as genetic model include investigations on C4 photosynthesis, domestication processes and mechanisms involved in abiotic stress tolerance (Li and Brutnell, 2011). *S. viridis* and *S. pumila* have, like *Arabidopsis* for dicotyl plants, other desirable characteristics including a rather small plant size (10–15 cm), a short life cycle (6–9 weeks) and they produce a high number of seeds. Furthermore, these species adapt well to very different conditions and tolerate well drought and cold, which makes them interesting models to study plant-microbe interactions. Surprisingly, despite their proven potential as model organisms, the microbiome of *Setaria* species has rarely been addressed in the literature. In this work, we surveyed and dissected the bacterial assemblages of surface-sterilized roots and panicles of wild *S. pumila* and *S. viridis* collected from 20 different sampling locations by 16S rRNA gene-based Illumina sequencing and furthermore genotyped plant populations. Our main objective was to gain understanding of the factors shaping the microbiota associated with each of these plant tissues, particularly of disseminative organs (ripe panicles harboring mature seeds), which have been rarely investigated. In addition, our aim was to analyze specific characteristics of root and panicle-associated microbiota and to obtain initial insight on the origin of bacteria associated with disseminative organs.

## Materials and Methods

### Sampling of *Setaria* Plants From 20 Locations in Austria

Whole plants were collected at the grain ripening stage (BBCH-89, Zadoks et al., 1974) from 20 sites in Austria with diverse soil and topographic characteristics (July/August 2013; Supplementary Figure S1 and Supplementary Table S1a). In total, 51 and 33 plants were collected for *S. viridis* and *S. pumila*, respectively, and all samples were collected in triplicates. Simultaneous sampling of both plant species was possible in locations B1, L4, L5, L9, S2, S3, S5, and V2. Additionally, 500 g bulk soil from each location were sampled and analyzed for their chemical parameters and clay content by the Austrian Agency for Health and Food Safety (AGES GmbH) (Supplementary Table S1b).

### Surface Sterilization and DNA Extraction From Plant Material

Whole roots and panicles were cut from each plant and washed with sterile water containing 0.1% Tween-20 for 10 min with shaking. Surface sterilization of each sample was achieved by immersion in a 5% sodium hypochlorite solution containing 0.1% Tween-20 for 5 min, followed by a 5-min incubation in 70% ethanol, and three subsequent washes with sterile distilled water. This treatment was performed twice. Microbial persistence on treated material was controlled by pressing on 10% TSA plates, which were subsequently incubated at 28°C for 5 days. No microbial growth was observed. In addition, presence of microbial DNA was tested by PCR amplification (see below) using 5 μl of the last washing water as template. No amplicons were obtained compared to a positive control which contained 12.5 ng of bacterial DNA as a template.

For total DNA extraction 0.2–0.5 g surface sterilized roots and panicles were air-dried under a laminar air flow and transferred to a 2 ml tube equipped with two sterile stainless-steel beads (5 mm) and immediately frozen in liquid nitrogen. Pulverization of the frozen material was achieved employing a mixer mill (Type MM301, Retsch) at a speed of 20 Hz for 30 s. The obtained frozen powder was immediately suspended in the Matrix E solution of the FastDNA^®^ SPIN Kit for Soil and the DNA extraction was performed following the manufacturer’s indications (MP Biomedicals). Quality and quantity of the isolated total DNA was confirmed by gel electrophoresis.

### Plant Genotyping by Genetic Diversity-Focused Genotyping by Sequencing (gd-GBS)

In order to explore the genetic diversity of the collected *Setaria* samples, the bench protocol presented by Peterson et al. (2014) was employed: Briefly, 200 ng of plant genomic DNA were digested with two restriction enzymes (*Pst*I and *Msp*I), and the resulting fragments were ligated to a pair of enzyme-specific adapters. After ligation, fragments were amplified with adapter-specific primers containing barcodes and the flow cell annealing (FCA) complementary sequences required for sequencing on the Illumina-MiSeq platform. The resulting amplicons were then pooled in equimolar amounts. Amplicons between 400 and 600 bp were subsequently excised from an agarose gel and used for further library assembly (Peterson et al., 2014). Sequencing was done at AIT using 2 × 300 bp Illumina MiSeq v3 sequencing (Illumina, San Diego, CA, United States). Obtained FASTQ files were loaded into the npGeno pipeline (Peterson et al., 2014) for SNP calling of each *Setaria* species separately, allowing 5% of the loci to contain missing values. The number of clusters of individuals for each sampled *Setaria* species was determined using the STRUCTURE software (Pritchard et al., 2000) and running the simulation for *K*-values from 1 to 10 using the following parameters: Length of Burnin Period = 10000, number of MCMC Repetitions after Burning = 50000. Estimation of K (number of clusters of individuals) for each sampled *Setaria* species was performed using the Structure Harvester online tool (Earl and vonHoldt, 2012) utilizing the DeltaK method (Evanno et al., 2005). A Maximum parsimony tree was generated in MEGA6 (Kumar et al., 2016) and edited in iTOL v3.4 (Letunic and Bork, 2007).

### Generation of 16S rRNA Gene Amplicon Libraries

Bacterial communities of roots and panicles were assessed by sequencing amplicons of the V5–V7 region of the 16S rRNA gene, obtained by two rounds of PCR amplification with a high-fidelity polymerase (KAPAHiFi^TM^ PCR Kit, KAPA Biosystems) and employing a strategy to overcome mitochondrial DNA interference (Supplementary Method S1). All amplicons were purified with the Agencourt^®^ AMPure^®^ XP system and quantified with Quant-iT^TM^ PicoGreen^®^ following the indications of the manufacturers. Purified amplicons were subsequently pooled in equimolar amounts and the quality of the library was assessed by 2100 Bioanalyzer (Agilent Technologies). Libraries of both roots and panicles were subjected to Illumina-adapter ligation and sequencing using 2 × 250 bp MiSeq v2 sequencing (Illumina Inc., San Diego, CA, United States) at LGC Group (Berlin, Germany).

### Raw Sequence Data Processing

MiSeq raw data quality was checked in FastQC (Andrews, 2010) and reads were screened for PhiX contamination using Bowtie 2.2.6 (Langmead and Salzberg, 2012). Reads were demultiplexed with a Maximum likelihood approach (Renaud et al., 2015) and primers were then stripped employing Cutadapt 1.8.3 (Martin, 2012). A Bayesian clustering for error correction was applied (Nikolenko et al., 2013; Schirmer et al., 2015) before merging the PE reads using PEAR 0.9.6 (*p* < 0.001) (Zhang et al., 2014). A quality filtering was performed in USEARCH v8.0.1517 (maximum expected error = 0.5) (Edgar, 2013; Edgar and Flyvbjerg, 2015). METAXA2 was used to target the extraction, verify and orient the 16S V5–V7 region of the filtered sequences (Bengtsson-Palme et al., 2015). Targeted reads were labeled according to the sample name of origin and combined in QIIME (Caporaso et al., 2010). Sequences were de-replicated, sorted and clustered at 97% of similarity using VSEARCH 1.1.1 (Rognes et al., 2016). Chimeras were checked adopting both a *de novo* and a reference-based approach, as routine of the above-mentioned tool. The RDP classifier training set v15 (Edgar and Flyvbjerg, 2015) was used as a database for the reference-based chimera detection. An optimal global alignment was applied in VSEARCH and a BIOM table generated. Taxonomy assignment was performed employing the naïve Bayesian RDP classifier v2.10 (Wang et al., 2007) in QIIME with a minimum confidence of 0.8 against the SILVA database version 132 (Quast et al., 2013; Schirmer et al., 2015).

### Bacterial Community Analyses

Sequence data are available at NCBI SRA database under the accession SRP145161 and the BioProject number PRJNA470571. Alpha-diversity values were calculated using the rtk R package (Saary et al., 2017) after multiple rarefactions, averaging the results of 9999 iterations. For beta-diversity purposes, a Cumulative Sum Scaling (CSS) normalization (Paulson et al., 2013) was applied. We used the Simpson’s index to quantify bacterial diversity. Differences in bacterial community composition across sample types were assessed by calculating pairwise Bray–Curtis dissimilarities from Hellinger-transformed values of relative abundances. Principal coordinate analysis was used for visualization of differences in β-diversity between sample types (plant organ and plant species). The effect of sampling location was assessed using a subset of the data containing samples from those locations where both plant species were collected simultaneously (locations B1, L4, L5, L9, S2, S3, S5, and V2). For this, relative abundances of OTUs within each subset was used for calculation of Bray–Curtis dissimilarity matrix and hierarchical clustering, and subsequently visualized with the heatmap.2 function of the gplots R-package. In order to identify tissue-specific and shared microbiota among sample types, reproducibly occurring OTUs (rOTUs), described as those OTUs present in at least two of three replicates were extracted from the data set and submitted to the jvenn online tool (Bardou et al., 2014). Identification of differential OTU abundances in panicle tissues was conducted employing the DESeq function of the DESeq2 R-package (Love et al., 2014) using raw root OTU counts as reference. To this end, samples with less than 500 OTU counts were removed from the data set and the adjusted *P*-value was set to a cutoff of 0.01. We fitted a linear model to describe the relationship between rOTUs occurrence and the log of their abundances for each plant compartment.

### Statistical Analyses

Processed sequence data were analyzed in R v3.5.1 software environment using the Phyloseq (McMurdie and Holmes, 2013) and vegan (Oksanen et al., 2018) packages. We assessed statistical significance at α = 0.05 (unless differently indicated) and, whenever necessary, adjusted *P*-values for multiple comparisons using false discovery rate controlling procedures. Richness and diversity across organ and plant species were compared using linear mixed-effect models with sampling location as a random factor. The effect of all factors (organ, plant species, and sampling location) in the alpha diversity measures were assessed by fitting a linear model on a subset of the data that contained exclusively samples from locations B1, L4, L5, L9, S2, S3, S5, and V2. Both analyses were followed by an analysis of variance (ANOVA). Additionally, differences between levels in each factor were further investigated in a *post hoc* analysis employing an Estimated Marginal Mean (EMM) approach (Lenth, 2018). Effects of each factor in the community composition (beta diversity) were tested by permutational multivariate analysis of variance (PERMANOVA) using the same subset mentioned above. Pairwise comparisons among levels were conducted using the permwise.perm.manova of the RVAideMemoire package (Hervé, 2018). For constrained analyses, the vegan::capscale function was used, as well as the multiconstrained function of the BiodiversityR package for pairwise comparisons (Kindt and Coe, 2005). We verified that the data met the assumption of multivariate homogeneity of dispersions, which was conducted using the vegan::betadisper function before running these tests. Finally, to test whether compositional differences among sample types are correlated across plant organs, a Procrustes analysis was conducted employing the vegan: protest function. For this purpose, samples were split according to the plant organ and PCoA and CAP ordinations where calculated again with the criteria mentioned above.

## Results

### Bacterial Endophytic Structures and Diversity Among Plant Tissues

Sequencing of the V5–V7 region of the 16S rRNA gene from root and panicle samples yielded 7,132,407 high-quality merged reads, corresponding to an average of 42,455 ± 16,628 reads per sample, with an average read length of 384 bp. Root samples yielded a total of 3,711,149 merged reads, with an average of 44,180 ± 13,364 sequences per sample. This corresponded to an average of 229 ± 109 observed OTUs per sample. Panicle samples gave a total of 3,421,258 reads, with an average of 40,729 ± 19,280 reads and 72 ± 53 OTUs per sample. Read numbers were rarefied to 15,157 for each sample. From all 168 samples, 4483 OTUs were obtained. When the relative abundance of OTUs per sample was considered, the number of OTUs with a maximum relative abundance greater than 0.01% in at least one sample was 4049.

**FIGURE 1:**
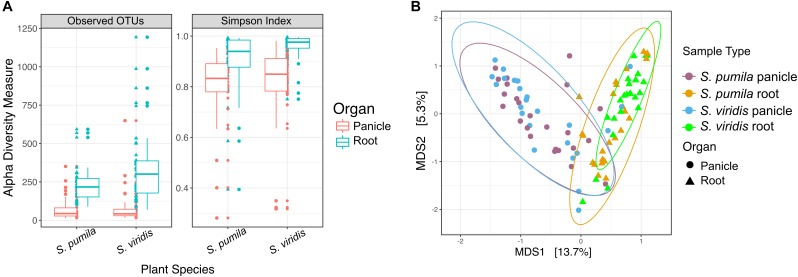
Bacterial endophytic diversity within roots and panicles of *Setaria pumila* and *Setaria viridis* **(A)** α-diversity metrics for communities within roots and panicles of *Setaria* spp. Significant (*p* < 0.001) higher richness was observed in root tissues when compared to panicle communities. Evenness (Simpson-index), was also significantly different between plant organs. **(B)** Principal coordinate analysis (PCoA) of *S. pumila* and *S. viridis* samples that were collected simultaneously from the same locations revealed the plant organ as the main driver of endophyte communities (separation across the first PC).

Bacterial richness (total observed OTUs) and evenness were significantly higher in roots than in panicle tissues (*p* < 0.001) in both *S. viridis* and *S. pumila*. (Figure 1A) and across sampling locations (Supplementary Table S3a). Differences in alpha diversity across plant species (*P* < 0.01) showed significantly higher richness but lower diversity values in *S. viridis* than in *S. pumila* samples. A slight but significant effect of the sampling location in the bacterial richness was also observed, albeit less pronounced than the effects of the organ and the plant species (Supplementary Tables S3b,c).

Moreover, the overall variation in bacterial community composition (β-diversity) was explained significantly and, in greater part, by the plant organ (*R2* = 0.19, *p* = 0.0001), followed by the sampling location (*R2* = 0.18, *p* = 0.0001) and lastly by the plant species (pseudo-*F* = 0.017, *p* = 0.0048) (Supplementary Tables S3d,e). The impact of the plant organ on shaping the beta diversity structures was confirmed by Procrustes analysis both applied to PCoA and CAP ordinations. The superimposition of patterns based on panicle samples over root samples showed that despite a significant correlation (*p* < 0.05), the degree of concordance was not strong, as indicated by a Pearson correlation coefficient *r* = 0.3 and a derived goodness of fit *m*_12_ = 0.9 (1-r^2^). Communities within roots and panicles were significantly different in composition (*p* < 0.001), regardless of the plant species (Figure 1B). Pairwise comparisons among all sample types revealed significant differences between the root microbiota of both *Setaria* species (*p* < 0.01) and between root microbiota and their panicle counterparts (*p* = 0.001). Interestingly, panicle microbiota of *S. viridis* and *S. pumila* did not show significant differences (*p* > 0.05) but revealed to be highly variable (Supplementary Figure S2 and Supplementary Table S4). Furthermore, a significant effect of the sampling location on both root and panicle communities for each *Setaria* species (*p* < 0.001) was observed (Supplementary Figure S3).

**FIGURE 2:**
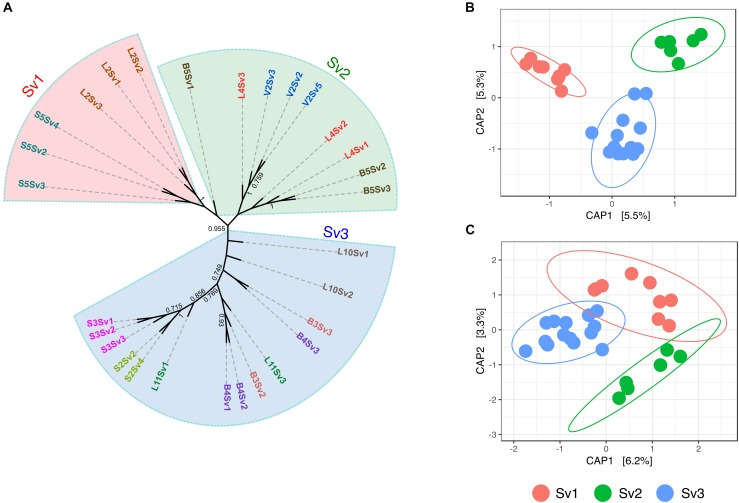
Assessed genotype groups of *S. viridis* samples and the relevance of these in the bacterial endophyte communities of roots and panicles **(A)** Maximum parsimony tree show the relatedness of the *S. viridis* samples enclosed by genotype groups Sv1, Sv2, and Sv3. Principal coordinate analysis (PCoA) biplot of the microbial communities of **(B)** roots and **(C)** panicles of *S. viridis.* PCoA were constrained to genotype groups.

To assess the role of plant genotype at an intra-species level, the gd-GBS protocol presented by Peterson et al. (2014) was employed. In total, 29 samples of each plant species (Supplementary Table S5) delivered enough high-quality reads for further processing with the computational pipeline npGeno (Peterson et al., 2014). Overall, npGeno yielded 355 and 1402 SNPs for *S. pumila* and *S. viridis*, respectively. Simulation analyses revealed three clusters (*K* = 3) enclosing *S. viridis* individuals (Supplementary Figure S4), and one cluster (*K* = 1) for *S. pumila* samples, meaning no significant genetic diversity (not shown). For the three *S. viridis* genotypes, 6, 9 and 14 samples were attributed to each of the three identified genotype sub-groups (Sv1, Sv2, and Sv3), respectively, (Figure 2A). Root bacterial communities of each of the genotype subgroups were significantly different in composition (*p* < 0.001) (Figure 2B). In contrast, panicles samples belonging to Sv3 showed significantly different community structures (*p* < 0.05) to those of Sv1 and Sv2 (*p* = 0.3) (Figure 2C).

### Taxonomic Composition of Bacterial Assemblages in *Setaria*

Overall, 4483 OTUs were assigned to 79 bacterial classes within a total of 41 phyla. Root and panicle samples of both *S. viridis* and *S. pumila* showed similar taxonomic compositions at phylum level (Supplementary Figure S5a). *Proteobacteria* was the most abundant phylum within roots and panicles of both *Setaria* species, followed by *Actinobacteria, Bacteroidetes, Saccharibacteria* (formerly TM7), and *Firmicutes*. Altogether, these five phyla contributed to 87% of the obtained reads of this data set (Supplementary Figure S5b). At the class level, bacterial assemblages were dominated by *Gammaproteobacteria*, with additional highly represented classes like *Alphaproteobacteria, Deltaproteobacteria, Actinobacteria, Betaproteobacteria*, and *Sphingobacteriia* which were widespread among roots and panicles.

**FIGURE 3:**
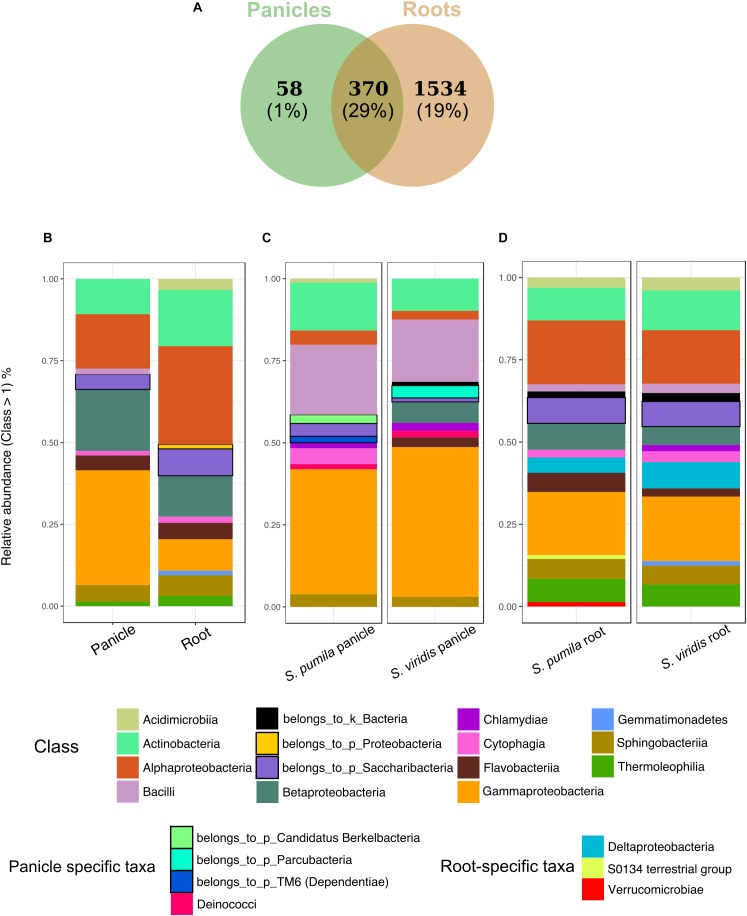
Bacterial endophytic communities of root and panicle samples. **(A)** Numbers of rOTUs in roots and panicles and their contribution to the overall read count (%). **(B)** Taxonomic structures of the shared communities (*n* = 370) in each plant organ. **(C)** Taxonomic distribution of panicle-specific communities (*n* = 58) for each plant species. **(D)** Taxonomic distribution of root-specific communities (*n* = 1534) for each plant species.

In order to identify taxonomic differences and similarities across sample types, a subset of the data set enclosing rOTUs was generated (see also Pfeiffer et al., 2017). We identified 1962 rOTUs which contributed to 49% of the obtained reads and represented a total of 30 bacterial phyla. Roots hosted more diverse communities with 1904 rOTUs, whereas 428 rOTUs of 20 classes were found in panicle samples. In total, 370 rOTUs of 29 bacterial classes were observed in both root and panicle tissues (Figure 3A). However, within these shared communities relative abundances of bacterial taxa differed in each plant organ. For instance, rOTUs assigned to *Gammaproteobacteria* summed up to 33% of the reads in the panicles, with *Enterobacteriaceae* contributing to more than 16%. Conversely, it was the *Alphaproteobacteria* rOTUs which dominated in root samples, with the order *Rhizobiales* contributing to more than 13% (Figure 3B and Supplementary Data S1).

Additionally, organ-specific rOTUs were identified. Panicle-specific communities enclosed 58 rOTUs which were dominated primarily by *Gammaproteobacteria* (mainly represented by *Enterobacteriaceae* and *Moraxellaceae*). Also common were *Bacilli* (*Paenibacillaceae*) as well as *Actinobacteria* (Figure 3C). Furthermore, ten genera were observed uniquely within panicle samples, namely *Achromobacter, Brevibacterium, Candidatus* sulcia, *Enhydrobacter, Ornithinimicrobium, Pseudoxanthomonas, Tepidimonas, Thermicanus, Thermus*, and *Turicella.* Conversely, root-specific communities were highly diverse, harboring 1534 rOTUs. Here, the order *Legionellales* was the most represented amongst the dominant *Gammaproteobacteria*, with *Legionella* and *Aquicella* summing up to 12% of the root-specific reads (Figure 3D). Root-specific communities were also highly represented by the order *Rhizobiales*.

We further compared the communities associated to *S. pumila* and *S. viridis* plants from locations B1, L4, L5, L9, S2, S3, S5, and V2, as it was possible to collect samples of both *Setaria* species from these sites. A total of 536 rOTUs were present in both plant species (Supplementary Figure S6). Overall, *S. viridis* samples showed a higher richness and diversity of bacterial classes compared to *S. pumila* (Supplementary Figure S6a). Plant species-specific rOTUs were identified, however, community composition based on the relative abundances of predominant bacterial classes was similar between both *S. viridis* and *S. pumila* (Supplementary Figure S6b).

### Root and Panicle Microbiota of *Setaria*

**FIGURE 4:**
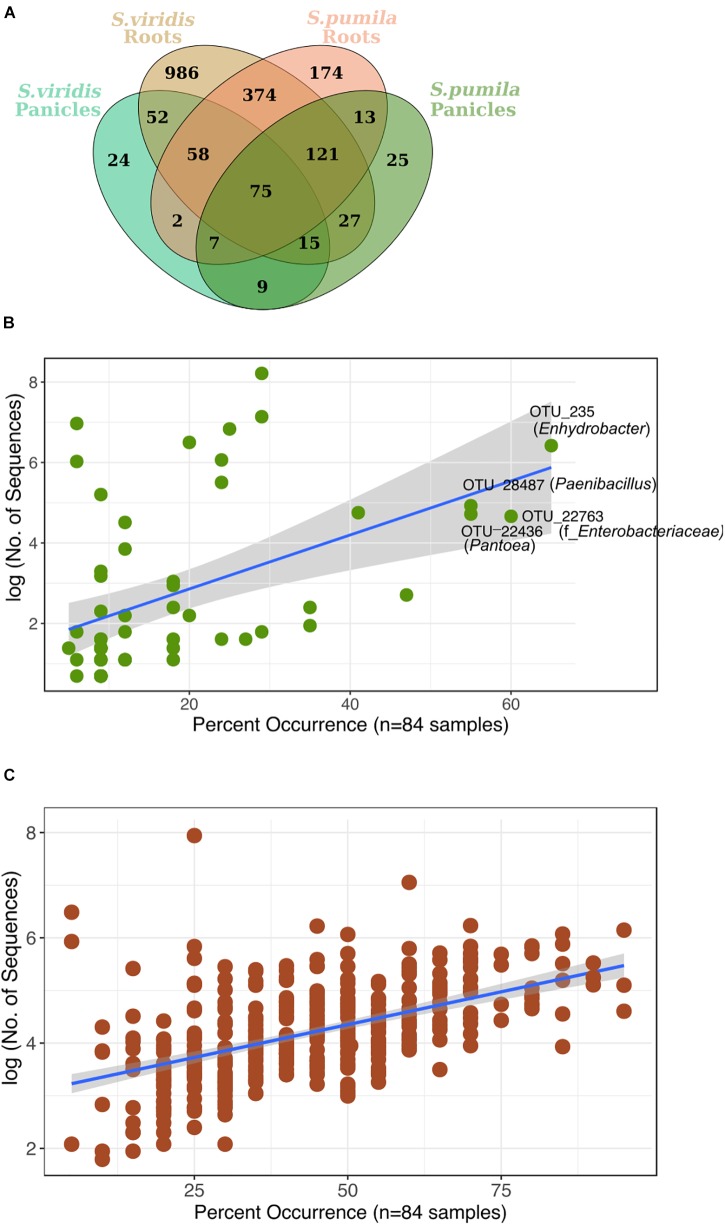
**(A)** Reproducibly occurring rOTUs in all sample types. **(B)** Panicle-specific rOTUs (*n* = 58) show relatively low occurrence percentage across sampling locations. The most prevalent members of the panicle communities occur in up to 65% of locations. **(C)** Root-specific rOTUs that were shared between both plant species (*n* = 374) contain a core-root microbiota which prevails in over 80% of all sampling locations. Each rOTU is a point, and the line shows the log-linear model for the occurrence/abundance relationship. The gray shading represents the standard error.

Reproducibly occurring OTUs for each sample type were further investigated based on their occurrences across samples of all locations (Figure 4A). Panicle-specific rOTUs were characterized by low occurrences across samples, ranging from 5 to 65% of all locations (Figure 4B). Altogether, panicle-specific assemblages of each *Setaria* species showed similarities in composition at the class level (Supplementary Figure S7). However, at lower taxonomic levels, communities differed between both *S. viridis* and *S. pumila* (Supplementary Figures S7b,c).

On the other hand, root-specific microbiota of each *Setaria* species were dominated by high abundances of *Legionalles* (*Legionella* and *Aquicella*) and *Saccharibacteria* (Supplementary Figures S8a,b), and showed relatively low occurrences across sampling locations (Supplementary Figure S8c). Further, a group of 374 root-specific rOTUs were found in both plant species and contained core root microbiota comprising 21 rOTUs, which were present in samples of up to 95% of all locations (Figure 4C). This core root microbiota was represented by 10 bacterial orders that were governed by *Rhizobiales*, and included representatives of the *Streptomycetales, Burkholderiales*, and *Sphingobacteriales* (Supplementary Figure S9).

**FIGURE 5:**
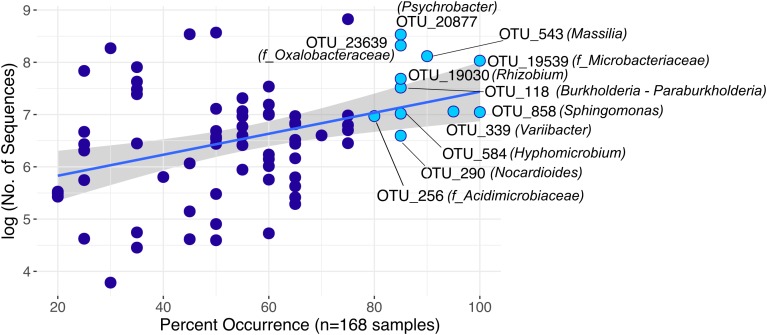
Highly distributed rOTUs (*n* = 75) across all sample types. Each rOTU is a point, and the line shows the log-linear model for the occurrence/abundance relationship. The gray shading represents the standard error. Highlighted rOTUs represent the core microbiota detected in samples of at least 80% of all locations.

Furthermore, we identified 75 rOTUs of 12 bacterial classes occurring in all sample types (Supplementary Figure S10). Among these, ten rOTUs were significantly enriched in panicles (Supplementary Table S6). Interestingly, the nucleotide sequence of OTU_22355 showed 99% identity to numerous entries of uncultured clones, including some detected in rice seed microbiomes (GenBank IDs: HQ610793 and HQ610771). When restricting the BLAST search results to contain only hits of cultured strains, much lower identity scores (up to 97%) for four of the *Enterobacteriaceae* rOTUs were obtained. Moreover, we observed an overall core microbiota of eleven rOTUs that occurred in samples from at least 80% of all locations and contributed to 4% of all read counts (Figure 5). Two of these core rOTUs were classified as *Psychrobacter* and *Massilia* and were also found significantly enriched in panicle tissues (Supplementary Figure S10). The complete list of differentially abundant rOTUs in panicles can be found in the Supplementary File Data S2.

## Discussion

### Tissue, Sampling Site and Plant Genotype Significantly Shape the *Setaria* Microbiota

The overall assemblages associated with roots and panicles of *S. pumila* and *S. viridis* were significantly shaped mainly by the plant organ, followed by the sampling site and lastly, by the plant species. The observation that different plant tissues harbor communities with distinct microbial numbers and phylogenies are consistent with previous studies (Kaga et al., 2009; Compant et al., 2011; Hameed et al., 2015), however, differences among root and inflorescence microbiota of the same plant have been rarely addressed (Aleklett et al., 2014). Divergences in microbiota associated with roots and panicles may be attributed to the sources and routes employed by the microbes colonizing each of these plant compartments. For instance, plant roots have been reported to be the most heavily colonized plant organ (Hallmann and Berg, 2006), since they are exposed to the vast microbial load and diversity immersed in the nutrient-rich and protected microenvironment of the rhizosphere (Bulgarelli et al., 2013). Several studies have demonstrated the systematic spreading of bacteria from the root endodermis to aerial plant compartments through, for example, the xylem system as observed in rice seedlings (James et al., 2002) and grapevine (Compant et al., 2008), which supports the overlap of rOTUs present in both roots and panicles in this study. However, only a small fraction of root-derived microorganisms may be able to colonize aboveground plant tissues, particularly disseminative organs (e.g., inflorescences and seeds), as they must possess the physiological requirements to establish in these niches (Okunishi et al., 2005; Compant et al., 2010; Aleklett et al., 2014). Moreover, bacteria from alternative sources may colonize reproductive and disseminative plant organs by using entry points along the external microenvironments of stems (caulosphere), flowers (anthosphere) or fruits (carposphere) (Compant et al., 2010; Hardoim et al., 2015; Mitter et al., 2017). Bacterial associations with gametes, like pollen grains, have also been described (Madmony et al., 2005; Fürnkranz et al., 2012; Ambika Manirajan et al., 2016), and may result in the colonization of the ovule and the resulting seed after pollination (Agarwal and Sinclair, 1996).

The plant species significantly explained part of the variation observed for the microbiota of roots which further reflected the genetic diversity and relatedness of their hosts at the intra-species level. Conversely, panicle microbiota of *S. viridis* and *S. pumila* did not show significant differences, but instead showed to be highly variable. Host effects are well known to shape root-associated microbial communities, as root exudates include a variety of compounds like sugars, organic acids, vitamins, hormones, and antimicrobials which are known to enrich or deplete potential root colonizers (Haichar et al., 2008; Turner et al., 2013; Edwards et al., 2015). Similarly, chemicals released by inflorescences (e.g., volatiles, sugars and lipids in stigmatic and pollen exudations) may play a vital role in the early assembly of communities associated with reproductive organs by allowing or deterring bacterial populations that may derive from rain and bioaerosols or those associated to visiting insects (Snoeren et al., 2007; Frago et al., 2012; Junker and Tholl, 2013; Aleklett et al., 2014; Lòpez-Fernàndez et al., 2017). Consequently, communities found in flowers of different plant species often show taxa rarely found in soils and other plant compartments (Junker et al., 2011; Shade et al., 2013; Aleklett et al., 2014) and may face selective pressures from a plethora of abiotic stresses including plant-derived substrates and compounds (reviewed by Aleklett et al., 2014; Burdon et al., 2018). Our results on the overall panicle microbiota indicate either no effect of compounds released by inflorescences, or most probably a higher dependence on the environment of the sampling location. Generally, chemical composition and amounts of exudates and volatiles can vary among plant species and cultivars (Mark et al., 2005; Micallef et al., 2009; Niederbacher et al., 2015), indicating that the assembly of at least part of the bacterial communities of both roots and inflorescences is not a purely stochastic process, but is rather largely restrained by the host plant genotype.

### The *Setaria* Microbiota

In this study, we addressed differences and similarities across plant tissues in order to understand the specific characteristics involved in the assembly of each of their endophytic communities, especially those within disseminative organs. Roots and panicles of *Setaria* spp. harbored communities that were similar at high taxonomic levels and were dominated by five main phyla, namely *Proteobacteria, Actinobacteria, Bacteroidetes, Firmicutes*, and *Saccharibacteria* (formerly TM7). Members of the former four phyla are widespread among endospheres of several plant species and are to a great extent cultivable (reviewed in Hardoim et al., 2015). In contrast, *Saccharibacteria* have been solely detected by culture-independent approaches until recently, as they were reported to comprise epibionts that depend on other bacteria for cultivation and basic cellular building blocks like nucleotides and amino acids (He et al., 2015; Starr et al., 2018). *Saccharibacteria* have been detected across multiple environments, including the rhizospheres of several plant species (Weinert et al., 2011; Peiffer et al., 2013; Johnston-Monje et al., 2016; Lee et al., 2016), in the phyllospheres of spinach seedlings (Lopez-Velasco et al., 2013), as endophytes in roots of sugarcane (Dong et al., 2018) and as one of the predominant taxa colonizing apple flowers (Shade et al., 2013), indicating high ecological versatility.

Panicle tissues showed higher abundances of *Gammaproteobacteria* in both, panicle-specific and shared communities with roots, mainly attributed to the *Enterobacteriaceae*. Members of this family have been frequently detected in or isolated from surface-sterilized plant material, including seeds (e.g., Hardoim et al., 2012; Sessitsch et al., 2012; Cope-Selby et al., 2016; Yang et al., 2017). Intriguingly, ten rOTUs that were present in root and panicle samples of both plant species were found to be significantly enriched within panicle tissues. Five of these rOTUs belong to the *Enterobacteriaceae* and included members that were distantly related to yet known cultured taxa. Significantly higher abundances of these *Enterobacteriaceae* rOTUs in ripe grain-harboring panicles compared to the roots may suggest enrichment within the grain. Therefore, it is possible that these rOTUs represent a group of highly conserved, yet uncultured seed-borne taxa that may have a fully endophytic lifestyle. Further experiments are needed to address this hypothesis and should include taxonomic markers that allow tracking of bacterial groups down to strain level.

Conversely, Alphaproteobacteria were found in higher abundances among root assemblages, with *Rhizobiales* as the predominant order. *Alphaproteobacteria* comprise a large group of prominent nitrogen-fixing and symbiotic genera such as *Rhizobium* and *Bradyrhizobium* (Hardoim et al., 2015), as well as species that have shown nitrogen fixation *in planta* like *Gluconoacetobacter diazotrophicus* in sugarcane (Sevilla et al., 2001) and *Azospirillum brasilense* in *Setaria* spp. (Okon et al., 1983; Pankievicz et al., 2015). The *Setaria* weed species group is one of the most successful terrestrial plants on earth, being able to colonize, adapt and endure a plethora of disturbed habitats in temperate, tropical and subtropical regions. Members of this species are endowed with the ability to tolerate high soil salinity, soil drought, tillage and extreme temperatures (Darmency and Dekker, 2011). Although this ability has been attributed to the vast genotypic and phenotypic biodiversity within the *Setaria* species, it is possible that associations with endophytic nitrogen-fixing symbionts aid in enduring survival in detrimental conditions.

### Core vs. Non-core Microbiota in Roots and Panicles of *Setaria* spp.

We further investigated reproducibly occurring microbiota based on their association with roots and panicles of both sampled *Setaria* species, particularly those found in at least 80% of all locations. Core root communities were dominated by members of the *Rhizobiales* as well as of other bacterial orders such as *Burkholderiales* and *Sphingobacteriales*. This goes in line with the findings of Jin et al. (2017), who observed a similar core microbiota composition in the rhizoplanes of *Setaria italica* plants from different geographic locations, hinting toward a tight symbiotic relationship between *Setaria* and members of these bacterial orders. In contrast, non-core root microbiota that were specific to each plant species showed low occurrences across samples of all locations as well as high relative abundances of *Legionellales* (*Aquicella* and *Legionella*) and *Saccharibacteria*. Members of the *Legionellales* order are known to cause diseases in humans and plants (Adeleke et al., 2001; Palusińska-Szysz and Cendrowska-Pinkosz, 2009; Bogas et al., 2015), but also have been reported to be plant-associated (Köberl et al., 2015; Cope-Selby et al., 2016) and are potentially transmitted by phloem-feeding insects (Lòpez-Fernàndez et al., 2017).

Overall, panicle-specific communities were characterized by high variability and low occurrence percentages across locations, as well as distinguished taxonomic compositions for panicles of each host species. Therefore, no panicle-specific core microbiota was observed. Our data show a minimal overlap of bacterial genera between panicles of *S. viridis* and *S. pumila*, suggesting that location-specific and random (but precisely timed) events may result in the establishment of bacterial communities that are unique for inflorescence microbiomes in *Setaria*. Panicles of *Setaria* species are protected by tough bristles that may trap potential microbial carriers like insects, air-borne particles and water droplets. However, colonization through open spikelets is limited by a narrow time frame, as anthesis happens only once per spikelet, lasts for short periods of time (60 min) and occurs only at night and when temperatures are low (Rizal et al., 2013). Colonization of panicle endospheres may also occur through pollinators or injuries caused by phloem-feeding insects (Aleklett et al., 2014). Although *Setaria* species are considered to be wind pollinated, Rizal et al. (2013) showed that potential pollinators were attracted to panicle tissues just prior to anthesis. Furthermore, the presence of rOTUs classified as insect endosymbionts like *Buchnera* in our data set suggests that *Setaria* samples in this study once served as host for phloem-feeding aphids.

We identified a set of 75 rOTUs that were present in roots and panicles of both *S. pumila* and *S. viridis*. Among these, eleven core rOTUs of eight bacterial genera were identified. Since these core rOTUs are present in both roots and seed-harboring panicles, the question arises: Which members of this core microbiota derive from the parent plant (transmission through the seed) and which portion derives from the surrounding environment (soil, airborne particles, or insects)? Several studies suggest vertical transmission of bacterial seed endophytes (Mukhopadhyay et al., 1996; Hardoim et al., 2012; Liu et al., 2012; Gagne-Bourgue et al., 2013), which would allow a plant with an established endophytic community to transfer bacteria to their progeny (Ferreira et al., 2008). Two core rOTUs classified as *Psychrobacter* and *Massilia* were also found to be significantly enriched in panicles, which may suggest enrichment within the seeds. These genera have been detected in surface sterilized seeds of rice, maize, soy, *Tylosema esculentum*, and *Crotalaria pumila* (López-López et al., 2010; Liu et al., 2012; Hameed et al., 2015; Chimwamurombe et al., 2016; Sánchez-López et al., 2018). Other fairly unexplored genera among the overall core microbiota in this study included *Hyphomicrobium* and *Variibacter*, both members of *Rhizobiales*. *Hyphomicrobium* was recently attributed diazotrophic activities in sugarcane roots (Dong et al., 2018), while no previous records were found about *Variibacter* in association with plants. Grasses are also known to associate with fungi of the *Clavicipitaceae*, which may colonize the whole plant systematically and can be transmitted via seeds (Saikkonen et al., 2002; Hardoim et al., 2015). Recent work has shown that seed endophytic fungi of the *Hypocreales* order harbor endohyphal bacteria where *Enterobacteriaceae, Burkholderiaceae*, and *Rhizobiales* among others were most common (Shaffer et al., 2016). Hence, it may be possible that these taxa are transmitted to the plant through association with fungal endophytes.

Overall, we could demonstrate that reproductive organs such as panicles host highly variable bacterial communities, which are significantly different to those found in roots and seem to derive only partly from the soil environment. The finding of core rOTUs that were widely distributed among sampling sites, indicates the presence of ubiquitous and highly adapted bacterial groups inhabiting *Setaria* tissues, or more intriguingly, suggests a conserved microbiota that perseveres across sampling locations, potentially through vertical transmission. Future insights in the function and relevance of these microbiota in the plant performance are yet to be elucidated and will serve as baseline for understanding the success of the *Setaria* species and its translation to the improvement of related agronomic crops.

## Author Contributions

CER contributed with laboratory work, data analyses, text writing, and result discussion. BM participated in sampling, experimental design, and discussion of the results. LA was involved in the data analysis. FT contributed to sampling, experimental design, and discussion of the results. SC was involved in the experimental design. AS contributed to experimental design, sampling, text writing, and discussion of the results.

## Conflict of Interest Statement

The authors declare that the research was conducted in the absence of any commercial or financial relationships that could be construed as a potential conflict of interest.
