# Supplementary material for: Roots and Panicles of the C4 Model Grasses Setaria viridis (L). and S. pumila Host Distinct Bacterial Assemblages With Core Taxa Conserved Across Host Genotypes and Sampling Sites
Source: Front Microbiol. 2018 Nov 12;9:2708. doi: 10.3389/fmicb.2018.02708 (PMC6240606; doi:10.3389/fmicb.2018.02708)
Supplement: Supplementary file 1 [file Data_Sheet_1.docx]

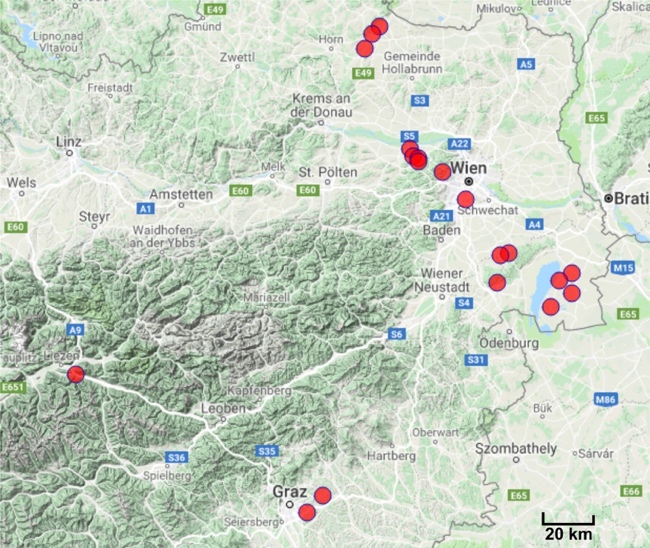


**Fig. S1.** Sites in Austria from which Setaria plants and soils were obtained in July-August 2013. Soil characteristics and sample information are listed in Table S1.

**Table S1**. Sample origin **(a)** and soil chemical properties **(b)** of each sampling site (Figure S1). Soil parameters were measured by the Austrian Agency for Health and Food Safety (AGES GmbH).

**(a)**

| **Region** | **Location Code** | **Location** | **GPS Coordinates** | **Sampled species** | **Elevation above sea level [m]** | **Description of site** |
| --- | --- | --- | --- | --- | --- | --- |
| **Burgenland (B)** | **B1** | Gols | 47.88975, 16.9121 | *S. viridis* and *S. pumila* | 120 | Roadside: Maize field |
|  | **B2** | Podersdorf | 47.86403, 16.85043 | *S. pumila* | 118 | Sugarbeet field |
|  | **B3** | Illmitz | 47.76905, 16.80451 | *S. viridis* | 117 | Vineyard |
|  | **B4** | Frauenkirchen | 47.81756, 16.91192 | *S. viridis* | 120 | Ruderal along road |
|  | **B5** | Eisenstadt | 47.85348, 16.52413 | *S. viridis* | 239 | Fallow |
| **Lower Austria (L)** | **L2** | Pulkau 1 | 48.66825, 15.8313 | *S. viridis* | 319 | Pumpkin field |
|  | **L3** | Pulkau 3 | 48.71838, 15.86949 | *S. pumila* | 285 | Maize field |
|  | **L4** | Retz | 48.74428, 15.90811 | *S. viridis* and *S. pumila* | 290 | Abandoned maize field |
|  | **L5** | Tulbinger Kogel | 48.28875, 16.11467 | *S. viridis* and *S. pumila* | 195 | Street side |
|  | **L6** | Katzelsdorf | 48.27552, 16.11209 | *S. pumila* | 281 | Meadow |
|  | **L7** | Chorherrn | 48.29468, 16.08461 | *S. viridis* | 171 | Maize field |
|  | **L9** | Tulln (AIT) | 48.32101, 16.06962 | *S. viridis* and *S. pumila* | 173 | Parking lot |
|  | **L10** | Seibersdof | 47.95230, 16.5409 | *S. viridis* | 183 | Roadside |
|  | **L11** | Mannerdorf | 47.95914, 16.5869 | *S. viridis* | 214 | Roadside |
| **Styria (S)** | **S1** | Kitzeck im Sausal | 46.77283, 15.46015 | *S. viridis* | 422 | Meadow |
|  | **S2** | Hart bei Graz | 47.0469, 15.52784 | *S. viridis* and *S. pumila* | 376 | Ruderal along road |
|  | **S3** | Höf Lembach | 47.10407, 15.61335 | *S. viridis* and *S. pumila* | 409 | Ruderal along road |
|  | **S5** | Selzthal | 47.53350, 14.32167 | *S. viridis* and *S. pumila* | 657 | Backyard |
| **Vienna (V)** | **V1** | Liesing | 48.14593, 16.35928 | *S. viridis* | 185 | Ruderal (High salt usage) |
|  | **V2** | Rieglerhütte | 48.24088, 16.23739 | *S. viridis* and *S. pumila* | 347 | Fallow |

**(b)**

| **Region** | **Location Code** | **pH (CaCl_2_)** | **Phosphor (P):CAL [mg/kg]** | **Potassium (K):CAL [mg/kg]** | **Total Nitrogen (N) [%]** | **Clay content [%]** | **Calcium (Ca): exchangable [% of sorbtion]** | **Magnesium (Mg): exchangable [% of sorbtion]** | **Potassium (K): exchangable [% of sorbtion]** | **Sodium (Na): exchangable [% of sorbtion]** | **Exchange capacity [cmol/kg]** | **Chloride (soluble in water) [mg/kg]** | **Sulfate (soluble in water) [mg/kg]** |
| --- | --- | --- | --- | --- | --- | --- | --- | --- | --- | --- | --- | --- | --- |
| **Burgenland (B)** | **B1** | 7.2 | 252 | 180 | 0.35 | 16 | 82.2 | 10.5 | 1.9 | 0.5 | 24.44 | 6 | 21 |
|  | **B2** | 7.5 | 53 | 254 | 0.36 | 16 | 80.8 | 16.5 | 2.4 | 0.3 | 26.87 | 22 | 17 |
|  | **B3** | 7.4 | 198 | 508 | 0.19 | 18 | 85.2 | 7.8 | 6.4 | 0.6 | 21.35 | 12 | 2 |
|  | **B4** | 7.7 | 19 | 110 | 0.11 | 20 | 75.9 | 21.4 | 1.9 | 0.7 | 17.25 | 34 | 2 |
|  | **B5** | 7.4 | 27 | 63 | 0.15 | 12 | 93.7 | 5.1 | 1.1 | 0.1 | 17.28 | 7 | 2 |
| **Lower Austria (L)** | **L2** | *NA* | *NA* | *NA* | *NA* | *NA* | *NA* | *NA* | *NA* | *NA* | *NA* | *NA* | *NA* |
|  | **L3** | 6.6 | 119 | 484 | 0.27 | 28 | 81.3 | 12.3 | 6.2 | 0.1 | 23.75 | 18 | 41 |
|  | **L4** | 7.4 | 106 | 399 | 0.19 | 28 | 86.3 | 8.4 | 5.2 | 0.1 | 22.94 | 7 | 26 |
|  | **L5** | *NA* | *NA* | *NA* | *NA* | *NA* | *NA* | *NA* | *NA* | *NA* | *NA* | *NA* | *NA* |
|  | **L6** | 7.1 | 19 | 243 | 0.43 | 22 | 84.7 | 11 | 1.8 | 2.5 | 43.20 | 21 | 2 |
|  | **L7** | *NA* | *NA* | *NA* | *NA* | *NA* | *NA* | *NA* | *NA* | *NA* | *NA* | *NA* | *NA* |
|  | **L9** | 7.6 | 31 | 94 | 0.14 | 22 | 92.4 | 5.7 | 1.7 | 0.2 | 18.62 | 5 | 19 |
|  | **L10** | 7.4 | 77 | 191 | 0.30 | 20 | 86 | 11.7 | 2 | 0.3 | 25.22 | 17 | 53 |
|  | **L11** | 7.5 | 23 | 193 | 0.20 | 30 | 86.1 | 10.2 | 2.2 | 1.5 | 25.33 | 21 | 2 |
| **Styria (S)** | **S1** | *NA* | *NA* | *NA* | *NA* | *NA* | *NA* | *NA* | *NA* | *NA* | *NA* | *NA* | *NA* |
|  | **S2** | 7.1 | 19 | 51 | 0.35 | 12 | 87.5 | 8.4 | 0.4 | 3.7 | 25.61 | 67 | 13 |
|  | **S3** | 7 | 123 | 238 | 0.33 | 12 | 92.8 | 4.8 | 2.4 | 0.1 | 25.98 | 22 | 2 |
|  | **S5** | 7.2 | 19 | 194 | 0.85 | 8 | 93.5 | 5.1 | 1.3 | 0.1 | 35.95 | 8 | 35 |
| **Vienna (V)** | **V1** | 7.6 | 30 | 310 | 0.21 | 18 | 84.3 | 4.9 | 4.4 | 6.3 | 19.22 | 7 | 19 |
|  | **V2** | 7.4 | 19 | 121 | 0.33 | 20 | 91.8 | 6.6 | 1.5 | 0.1 | 23.42 | 1 | 2 |

**Method S1:**

**16S rRNA gene amplicon PCR for root and panicle samples**

The first PCR round was performed with 12.5 ng extracted DNA, and PCR primers 799f_mod 6 (Hanshew *et al.*, 2013) and 1392r. Additionally, to reduce chloroplast DNA contamination, a blocking primer 799_Block was designed to bind specifically to chloroplast DNA and inhibit amplification through a phosphoryl-group at the 3’-end (Table S2a). Fifty µl PCR reactions contained 1X KAPA puffer, 1.5 µl 10 mM dNTPs, 1 µl of each 10 µM primer (799_mod6/1392r/799_block see Table S2 A), 2.5 µl DNA template (5 ng/µl) and 1 µl DNA polymerase. Reactions were held at 95°C for 5 minutes, followed by 25 cycles of amplification at 98°C (20 s), 59°C (30 s) and 72°C (30 s), with a final extension step at 72°C (3 min). To overcome mitochondrial DNA interference, the PCR products were loaded onto a 2% agarose gel (in sterile 1xTAE) and the 593 bp amplicons (not always visible) were separated from the interfering 1000 bp mitochondrial bands by electrophoresis for 90 minutes at 100 V. The 593 bp band of each sample was excised from the gel using the X-tracta gel extraction tools (Sigma Aldrich). The gel pieces containing the bacterial amplicons were placed into the top a sterile filter tip and placed inside an Eppendorf tube. The PCR product was regained by centrifugation (1000 rpm, 1 minute) of the assembled tube. After centrifugation, the filter-tip was carefully removed, and 2 µl of eluate were used for the next PCR. The second PCR round was performed with primers 799f/1175r carrying sample-specific barcodes. (Table S2b), with thermal cycling as described above with a modified annealing temperature of 48°C.**Table S2.** Primers used for the amplification of region V5-V7 of the 16S rRNA gene of root and panicle bacterial communities. Sample-specific barcodes are shown in red.

| **Primer name** | **Oligo Sequence (5' to 3')** |
| --- | --- |
| **a. 16S rRNA PCR round 1** | |
| 799f_mod6 | MGGATTAGATACCCKGGT |
| 1392r | ACGGGCGGTGTGTRC |
| 799f_Block | TGGGATTAGAGACCCCA/3Phos/ |
| **b. 16S rRNA PCR round 2** | |
| 799F_1 | ACAACCAGTTAACMGGATTAGATACCCKG |
| 799F_2 | NAACAGACCTTAACMGGATTAGATACCCKG |
| 799F_3 | NNACAAGGTCTTAACMGGATTAGATACCCKG |
| 799F_4 | NNNAAGTCTTCGTAACMGGATTAGATACCCKG |
| 799F_5 | ACATGAGGTTAACMGGATTAGATACCCKG |
| 799F_6 | NAAGCTCACTTAACMGGATTAGATACCCKG |
| 799F_7 | NNACGATACGTTAACMGGATTAGATACCCKG |
| 799F_8 | NNNAATGCGCTATAACMGGATTAGATACCCKG |
| 799F_9 | ACCTCATCTTAACMGGATTAGATACCCKG |
| 799F_10 | NAACAGCTCATAACMGGATTAGATACCCKG |
| 799F_11 | NNACTGTTGACTAACMGGATTAGATACCCKG |
| 799F_12 | NNNAGAGTTGCTTAACMGGATTAGATACCCKG |
| 799F_13 | ACCTTGACATAACMGGATTAGATACCCKG |
| 799F_14 | NACAACTGTGTAACMGGATTAGATACCCKG |
| 799F_15 | NNACTTAGCACTAACMGGATTAGATACCCKG |
| 799F_16 | NNNAATACGACCTAACMGGATTAGATACCCKG |
| 799F_17 | ACGATCGTATAACMGGATTAGATACCCKG |
| 799F_18 | NACCAATCAGTAACMGGATTAGATACCCKG |
| 799F_19 | NNAGGACTTGTTAACMGGATTAGATACCCKG |
| 799F_20 | NNNAGCTGAATCTAACMGGATTAGATACCCKG |
| 799F_21 | ACTCACTGTTAACMGGATTAGATACCCKG |
| 799F_22 | NAGAGCAATGTAACMGGATTAGATACCCKG |
| 799F_23 | NNAGTCATCCTTAACMGGATTAGATACCCKG |
| 799F_24 | NNNAGTAGCCTATAACMGGATTAGATACCCKG |
| 799F_25 | AGATAGCGATAACMGGATTAGATACCCKG |
| 799F_26 | NAACGGAACATAACMGGATTAGATACCCKG |
| 799F_27 | NNATATAGCCGTAACMGGATTAGATACCCKG |
| 799F_28 | NNNAGTGAACTCTAACMGGATTAGATACCCKG |
| 799F_29 | ATACGGACTTAACMGGATTAGATACCCKG |
| 799F_30 | NAGCTCCTTATAACMGGATTAGATACCCKG |
| 799F_31 | NNATGCGCATATAACMGGATTAGATACCCKG |
| 799F_32 | NNNAGTGCTTCATAACMGGATTAGATACCCKG |
| 799F_33 | ATCACCATGTAACMGGATTAGATACCCKG |
| 799F_34 | NAGCTTCAGTTAACMGGATTAGATACCCKG |
| 799F_35 | NNATGTACTGGTAACMGGATTAGATACCCKG |
| 799F_36 | NNNATCGGTAGTTAACMGGATTAGATACCCKG |
| 799F_37 | ATCGAACCTTAACMGGATTAGATACCCKG |
| 799F_38 | NAGGTACAACTAACMGGATTAGATACCCKG |
| 799F_39 | NNATTGGAGTGTAACMGGATTAGATACCCKG |
| 799F_40 | NNNAGACATTCCTAACMGGATTAGATACCCKG |
| 799F_41 | ATGCAACACTAACMGGATTAGATACCCKG |
| 799F_42 | NAGGTGTGTTTAACMGGATTAGATACCCKG |
| 799F_43 | ATGGTAACGTAACMGGATTAGATACCCKG |
| 799F_44 | NAGTCGATACTAACMGGATTAGATACCCKG |
| 799F_45 | ATTCACCTGTAACMGGATTAGATACCCKG |
| 799F_46 | NAGTTGAGCATAACMGGATTAGATACCCKG |
| 799F_47 | ATTGACACCTAACMGGATTAGATACCCKG |
| 799F_48 | NATACGTTGCTAACMGGATTAGATACCCKG |
| 1175R_1 | ACAACCAGTTACGTCRTCCCCDCCTTCCT |
| 1175R_2 | NAACAGACCTTACGTCRTCCCCDCCTTCCT |
| 1175R_3 | NNACAAGGTCTTACGTCRTCCCCDCCTTCCT |
| 1175R_4 | NNNAAGTCTTCGTACGTCRTCCCCDCCTTCCT |
| 1175R_5 | ACATGAGGTTACGTCRTCCCCDCCTTCCT |
| 1175R_6 | NAAGCTCACTTACGTCRTCCCCDCCTTCCT |
| 1175R_7 | NNACGATACGTTACGTCRTCCCCDCCTTCCT |
| 1175R_8 | NNNAATGCGCTATACGTCRTCCCCDCCTTCCT |
| 1175R_9 | ACCTCATCTTACGTCRTCCCCDCCTTCCT |
| 1175R_10 | NAACAGCTCATACGTCRTCCCCDCCTTCCT |
| 1175R_11 | NNACTGTTGACTACGTCRTCCCCDCCTTCCT |
| 1175R_12 | NNNAGAGTTGCTTACGTCRTCCCCDCCTTCCT |
| 1175R_13 | ACCTTGACATACGTCRTCCCCDCCTTCCT |
| 1175R_14 | NACAACTGTGTACGTCRTCCCCDCCTTCCT |
| 1175R_15 | NNACTTAGCACTACGTCRTCCCCDCCTTCCT |
| 1175R_16 | NNNAATACGACCTACGTCRTCCCCDCCTTCCT |
| 1175R_17 | ACGATCGTATACGTCRTCCCCDCCTTCCT |
| 1175R_18 | NACCAATCAGTACGTCRTCCCCDCCTTCCT |
| 1175R_19 | NNAGGACTTGTTACGTCRTCCCCDCCTTCCT |
| 1175R_20 | NNNAGCTGAATCTACGTCRTCCCCDCCTTCCT |
| 1175R_21 | ACTCACTGTTACGTCRTCCCCDCCTTCCT |
| 1175R_22 | NAGAGCAATGTACGTCRTCCCCDCCTTCCT |
| 1175R_23 | NNAGTCATCCTTACGTCRTCCCCDCCTTCCT |
| 1175R_24 | NNNAGTAGCCTATACGTCRTCCCCDCCTTCCT |
| 1175R_25 | AGATAGCGATACGTCRTCCCCDCCTTCCT |
| 1175R_26 | NAACGGAACATACGTCRTCCCCDCCTTCCT |
| 1175R_27 | NNATATAGCCGTACGTCRTCCCCDCCTTCCT |
| 1175R_28 | NNNAGTGAACTCTACGTCRTCCCCDCCTTCCT |
| 1175R_29 | ATACGGACTTACGTCRTCCCCDCCTTCCT |
| 1175R_30 | NAGCTCCTTATACGTCRTCCCCDCCTTCCT |
| 1175R_31 | NNATGCGCATATACGTCRTCCCCDCCTTCCT |
| 1175R_32 | NNNAGTGCTTCATACGTCRTCCCCDCCTTCCT |
| 1175R_33 | ATCACCATGTACGTCRTCCCCDCCTTCCT |
| 1175R_34 | NAGCTTCAGTTACGTCRTCCCCDCCTTCCT |
| 1175R_35 | NNATGTACTGGTACGTCRTCCCCDCCTTCCT |
| 1175R_36 | NNNATCGGTAGTTACGTCRTCCCCDCCTTCCT |
| 1175R_37 | ATCGAACCTTACGTCRTCCCCDCCTTCCT |
| 1175R_38 | NAGGTACAACTACGTCRTCCCCDCCTTCCT |
| 1175R_39 | NNATTGGAGTGTACGTCRTCCCCDCCTTCCT |
| 1175R_40 | NNNAGACATTCCTACGTCRTCCCCDCCTTCCT |
| 1175R_41 | ATGCAACACTACGTCRTCCCCDCCTTCCT |
| 1175R_42 | NAGGTGTGTTTACGTCRTCCCCDCCTTCCT |
| 1175R_43 | ATGGTAACGTACGTCRTCCCCDCCTTCCT |
| 1175R_44 | NAGTCGATACTACGTCRTCCCCDCCTTCCT |
| 1175R_45 | ATTCACCTGTACGTCRTCCCCDCCTTCCT |
| 1175R_46 | NAGTTGAGCATACGTCRTCCCCDCCTTCCT |
| 1175R_47 | ATTGACACCTACGTCRTCCCCDCCTTCCT |
| 1175R_48 | NATACGTTGCTACGTCRTCCCCDCCTTCCT |

**Table S3. (a) Alpha diversity measures of roots and panicles across sampling locations (b)** Roles of organ, plant species and sampling location in alpha diversity measures. **(c)** pairwise comparisons in OTU richness among sampling locations **(d)** Explained amount of variance in beta diversity for each factor. **(e)** P-values show significant pairwise differences among microbial communities across sampling locations

| **Plant species** | **Sampling location** | **Organ** | **Estimated Marginal Means (EMMs) for richness** | **Contrasts in OTU richness**  **(alpha= 0.05)** | **Estimated Marginal Means (EMMs) for diversity (Simpsons)** | | | **Contrasts in diversity**  **(alpha= 0.05)** |
| --- | --- | --- | --- | --- | --- | --- | --- | --- |
| ***S. pumila*** | **B1** | Root | 319.8 | a | 0.94 | a | | |
|  |  | Panicle | 106.3 | b | 0.79 | b | | |
|  | **L4** | Root | 294.8 | a | 0.94 | a | | |
|  |  | Panicle | 81.3 | b | 0.79 | b | | |
|  | **L5** | Root | 271.3 | a | 0.84 | a | | |
|  |  | Panicle | 57.75 | b | 0.69 | b | | |
|  | **L9** | Root | 276.1 | a | 0.88 | | a | |
|  |  | Panicle | 62.5 | b | 0.73 | | b | |
|  | **S2** | Root | 292.04 | a | 0.9 | | a | |
|  |  | Panicle | 78.5 | b | 0.75 | | b | |
|  | **S3** | Root | 361.4 | a | 0.78 | | a | |
|  |  | Panicle | 147.9 | b | 0.93 | | b | |
|  | **S5** | Root | 321.9 | a | 0.89 | | a | |
|  |  | Panicle | 108.3 | b | 0.75 | | b | |
|  | **V2** | Root | 325.8 | a | 0.93 | | a | |
|  |  | Panicle | 112.28 | b | 0.78 | | b | |
| ***S. viridis*** | **B1** | Root | 359.1 | a | 0.88 | | a | |
|  |  | Panicle | 81.28 | b | 0.74 | | b | |
|  | **L4** | Root | 334.1 | a | 0.89 | | a | |
|  |  | Panicle | 120.55 | b | 0.74 | | b | |
|  | **L5** | Root | 310.55 | a | 0.79 | | a | |
|  |  | Panicle | 97.0 | b | 0.64 | | b | |
|  | **L9** | Root | 315.31 | a | 0.83 | | a | |
|  |  | Panicle | 101.8 | b | 0.68 | | b | |
|  | **S2** | Root | 331.3 | a | 0.85 | | a | |
|  |  | Panicle | 117.76 | b | 0.7 | | b | |
|  | **S3** | Root | 400.7 | a | 0.88 | | a | |
|  |  | Panicle | 108.37 | b | 0.73 | | b | |
|  | **S5** | Root | 361.2 | a | 0.84 | | a | |
|  |  | Panicle | 147.63 | b | 0.64 | | b | |
|  | **V2** | Root | 365.1 | a | 0.88 | | a | |
|  |  | Panicle | 151.54 | b | 0.73 | | b | |

**(b)**

|  | **Estimated Marginal Means (EMMs) for Richness** | **Richness** | **Estimated Marginal Means (EMMs) for Diversity** | **Diversity**  **(Simpsons Index)** |
| --- | --- | --- | --- | --- |
| **Organ** | Root: 434.95 | F=291.38, p<0.001 | Root: 0.87 | F=41.37, p<0.001 |
|  | Panicle: 129.28 |  | Panicle: 0.74 |  |
| **Plant species** | S. pumila: 249.96 | F=9.85, p<0.01 | S. pumila: 0.83 | F=4.99, p<0.01 |
|  | S. viridis: 314.27 |  | S. viridis: 0.78 |  |
| **Sampling location** | - | F=2.87, p<0.05 | - | F=1.17, p=0.33 |

**(c)**

| **Sampling location** | **Estimated Marginal Means (EMMs) for Richness** | **Contrasts in OTU richness**  **(alpha= 0.05)** |
| --- | --- | --- |
| **B1** | 232.66 | ab |
| **L4** | 207.69 | ab |
| **L5** | 184.15 | a |
| **L9** | 188.91 | a |
| **S2** | 204.90 | ab |
| **S3** | 274.26 | b |
| **S5** | 234.77 | ab |
| **V2** | 238.68 | ab |

**(d)**

|  | **F.Model** | **R2** | **Pr (>F)** |
| --- | --- | --- | --- |
| Sampling location | 4.434 | 0.17738 | 0.0001 *** |
| Plant species | 3.062 | 0.01750 | 0.0048 ** |
| Organ | 33.031 | 0.18878 | 0.0001 *** |
| Sampling location:Plant species | 2.581 | 0.10326 | 0.0001 *** |
| Sampling location:Organ | 1.944 | 0.07778 | 0.0003 *** |
| Plant species:Organ | 1.628 | 0.00930 | 0.0805 |
| Sampling location:Plant species:Organ | 1.505 | 0.06020 | 0.0096 ** |

**(e)**

|  | **B1** | **L4** | **L5** | **L9** | **S2** | **S3** | **S5** |
| --- | --- | --- | --- | --- | --- | --- | --- |
| **L4** | 0.15939 |  |  |  |  |  |  |
| **L5** | **0.02369** | **0.04480** |  |  |  |  |  |
| **L9** | **0.00504** | **0.00093** | **0.00140** |  |  |  |  |
| **S2** | 0.15939 | 0.09695 | **0.02369** | **0.01307** |  |  |  |
| **S3** | 0.10125 | **0.04662** | **0.03080** | **0.00700** | 0.33730 |  |  |
| **S5** | **0.01120** | **0.01792** | **0.01120** | **0.00093** | **0.03982** | 0.06924 |  |
| **V2** | **0.03982** | 0.06924 | **0.02316** | **0.00093** | **0.03982** | 0.08680 | **0.03982** |


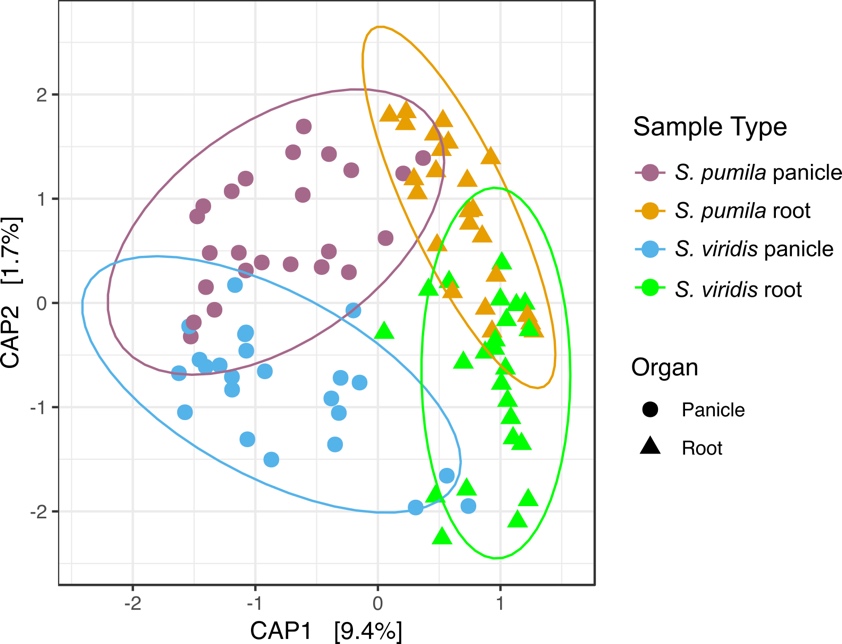


**Fig. S2.** Principal coordinate analysis (PCoA) of (a) roots and (b) panicles constrained to the plant species*.* Communities within panicles show a scattered pattern across the first principal component (CAP1), indicating that these samples are highly variable. A subset of the data containing samples that were collected simultaneously from the same locations was used.

**Table S4.** Pairwise comparisons of microbial community composition across sample types. The ordination is constrained to the plant species. A subset of the data containing samples that were collected simultaneously from the same locations was used.

| **Sample Type** | **P value** |
| --- | --- |
| *S. pumila* panicle vs. *S. pumila* root | 0.001 |
| *S. pumila* panicle vs. *S. viridis* panicle | 0.060 |
| *S. pumila* panicle vs. *S. viridis* root | 0.001 |
| *S. pumila* root vs. *S. viridis* panicle | 0.001 |
| *S. pumila* root vs. *S. viridis* root | 0.008 |
| *S. viridis* panicle vs *S. viridis* root | 0.001 |


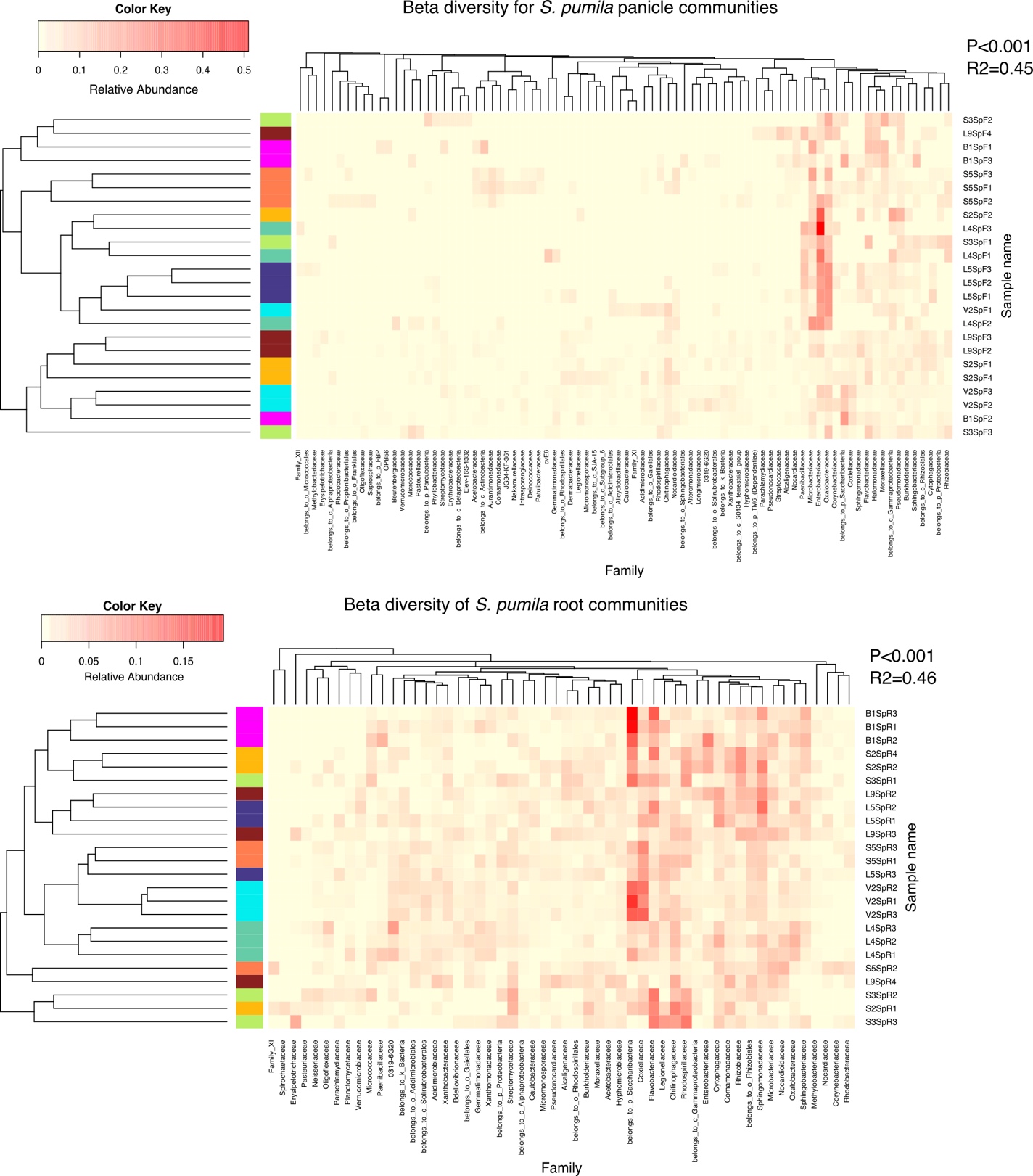


**Fig. S3.** Beta-diversity of bacterial endophytic communities of roots and panicles of S. viridis and S. pumila. Heatmaps are based on Bray-Curtis dissimilarity with subsequent hierarchical clustering of OTUs (x-axis) and sampling location (y-axis). Clusters of triplicates from each sampling location are displayed at the left side of each heatmap and indicated by different colors, based on the similarity of their microbial communities.


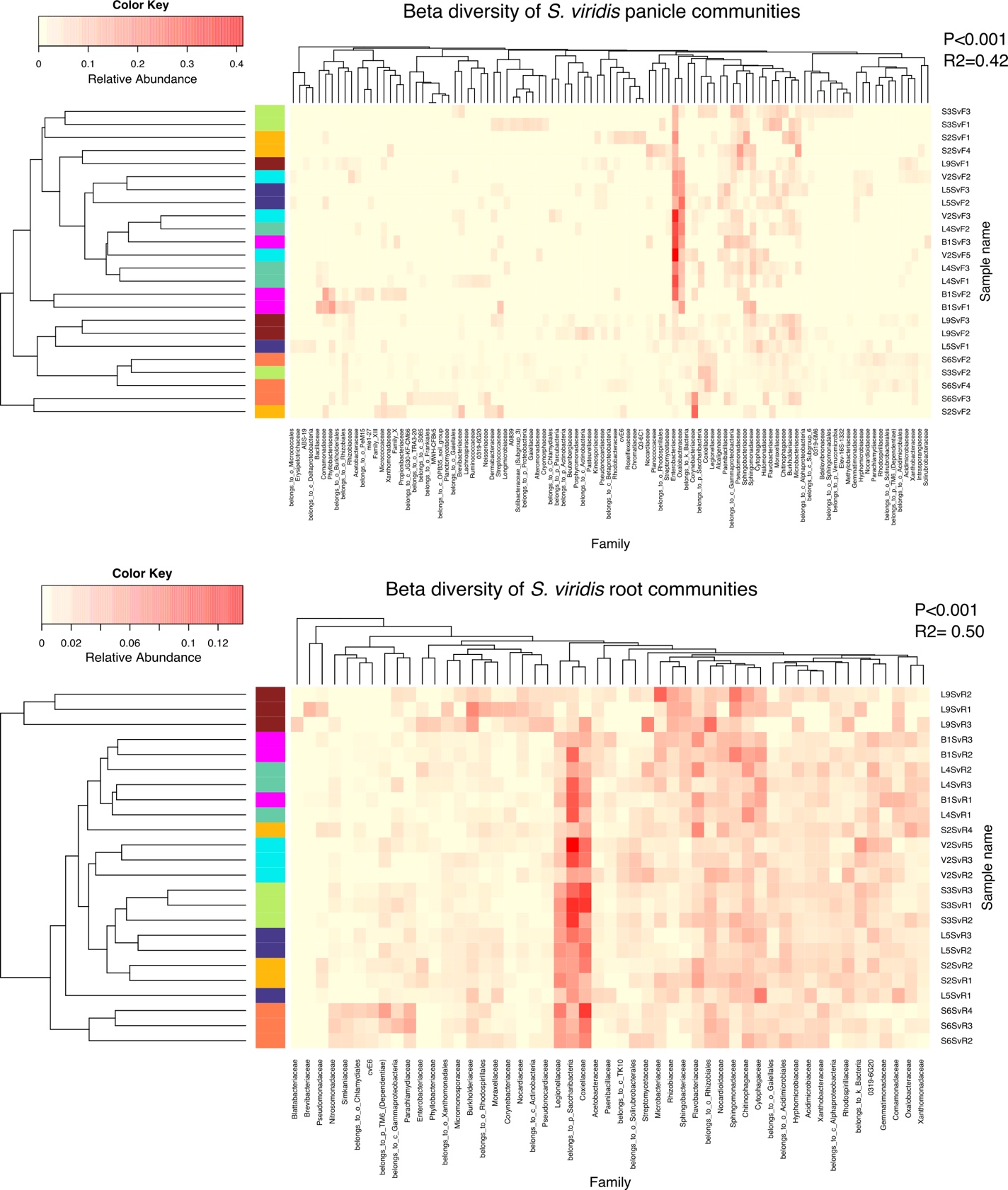


**Fig. S3. continued**

**Table S5.**  Sample names and the assigned number for the STRUCTURE analysis.

.


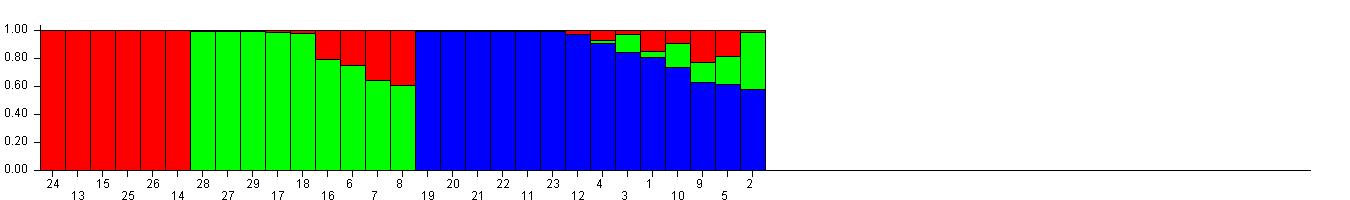


Sv1

Sv2

Sv3

**Fig. S4.** Optimal population structure (K=3) for the sampled *S. viridis* individuals. Each individual plant is represented by a number (Table S3) and single vertical bar, and each color represents one cluster of sub-populations. The length of each colored segment in a bar represents the estimated proportion of membership of an individual sample to a cluster as calculated by STRUCTURE


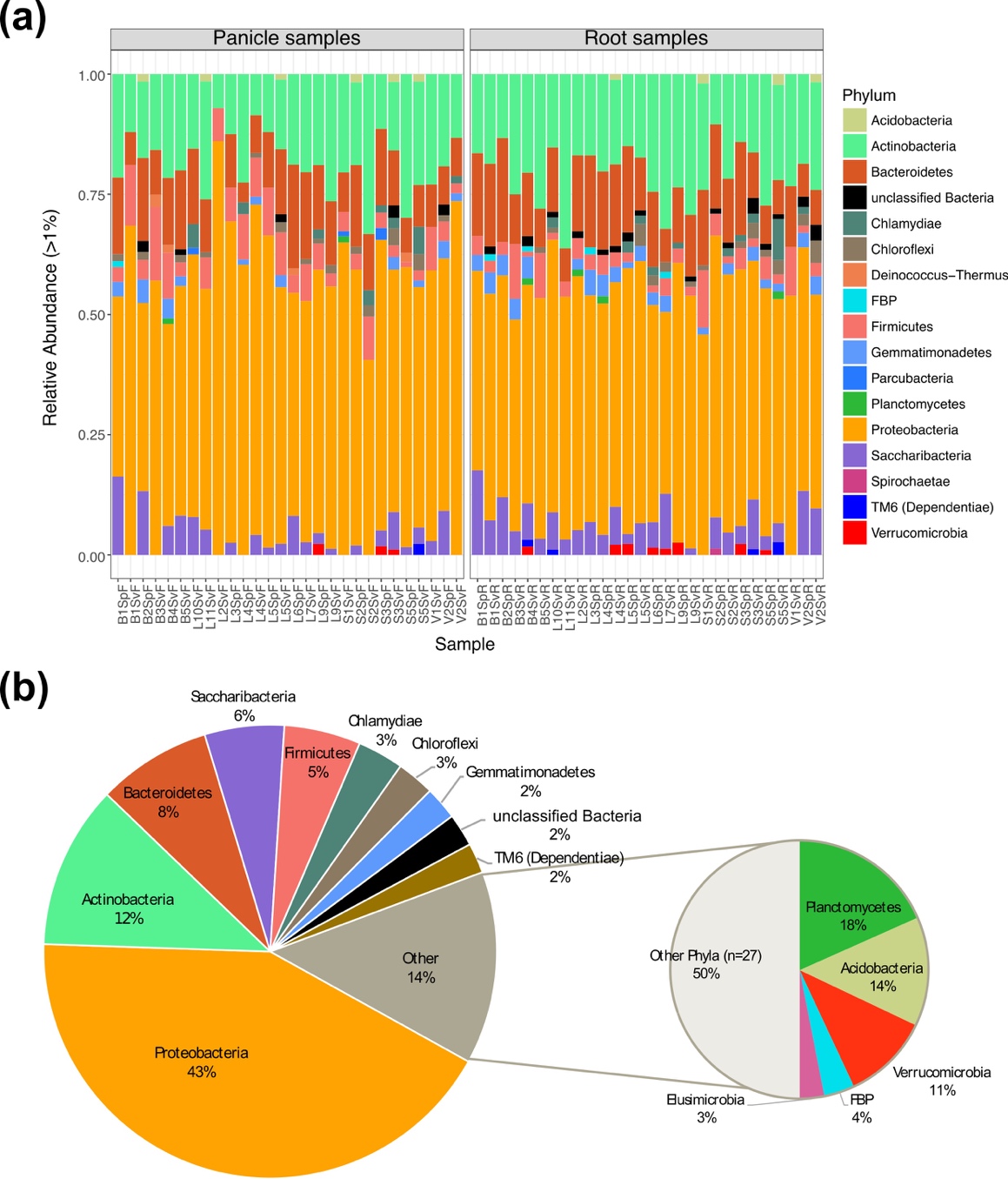


**Fig. S5.** Taxonomic distribution of bacterial endophytic assemblages of roots and panicles of S. pumila and S. viridis. **(a)** Relative abundances of the most abundant phyla (>1%) for merged triplicates of each location. **(b)** Dominance of bacterial phyla according to OTU numbers per taxon.


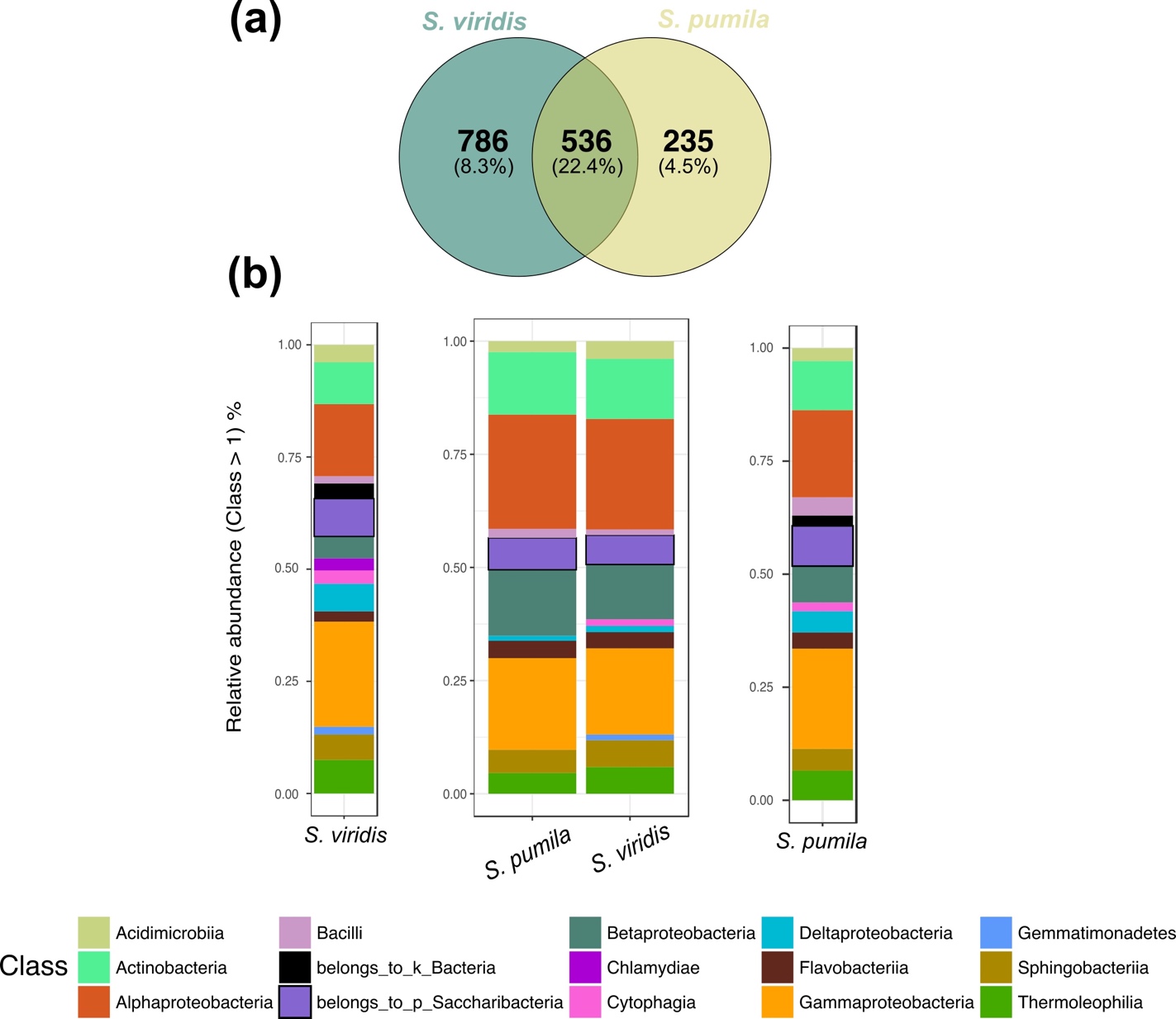


**Fig. S6.** Bacterial endophytic communities among samples of S. viridis and S. pumila. **(a)** numbers of rOTUs and their contribution to the overall read count (%) of S. viridis and S. pumila samples **(b)** Taxonomic structures of the communities found specifically in each plant species, as well as of shared assemblages


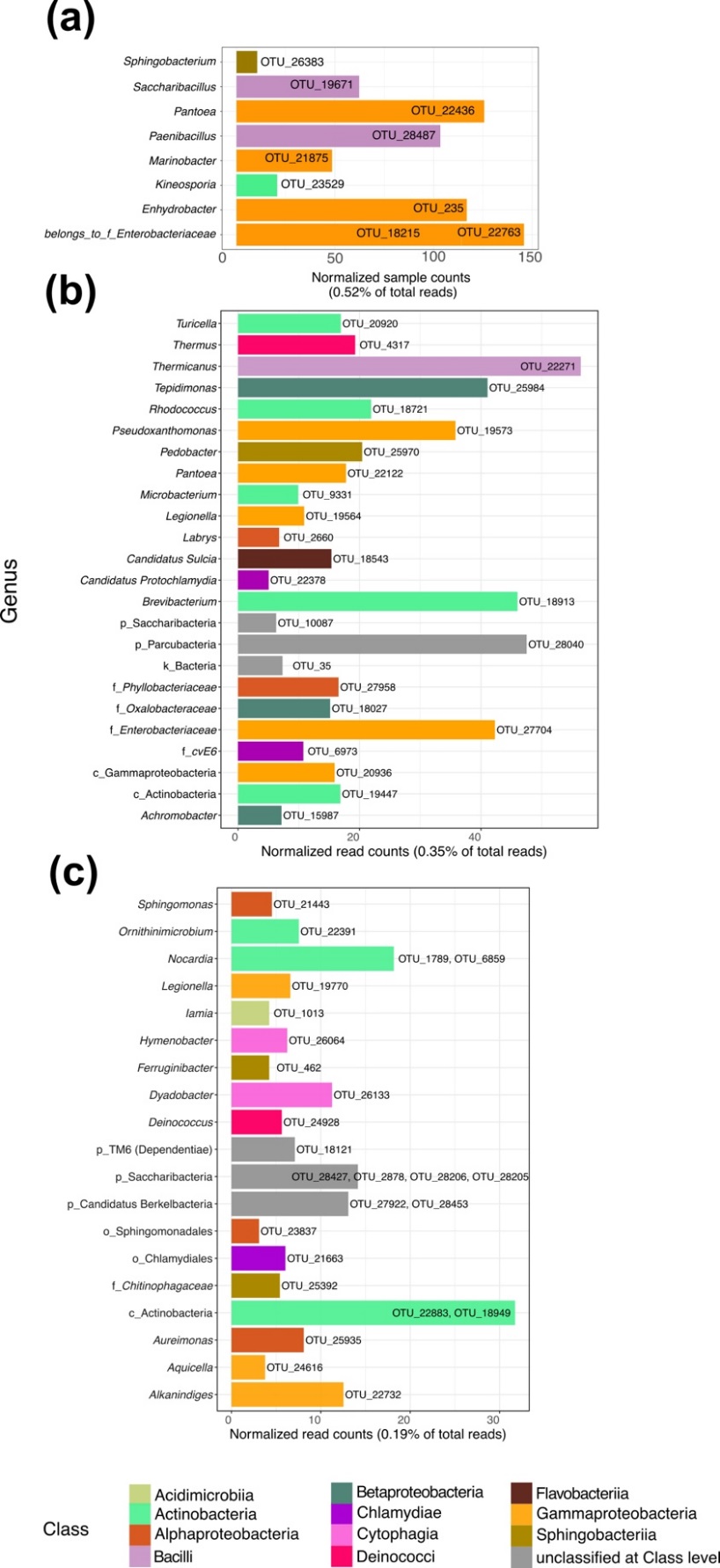


**Fig. S7.** Taxonomic composition of panicle-specific communities detected in (a) both *S. pumila* and *S. viridis* **(b)** *S. viridis* and **(c)** *S. pumila.* OTU read counts were normalized by cumulative sum scaling (CSS).


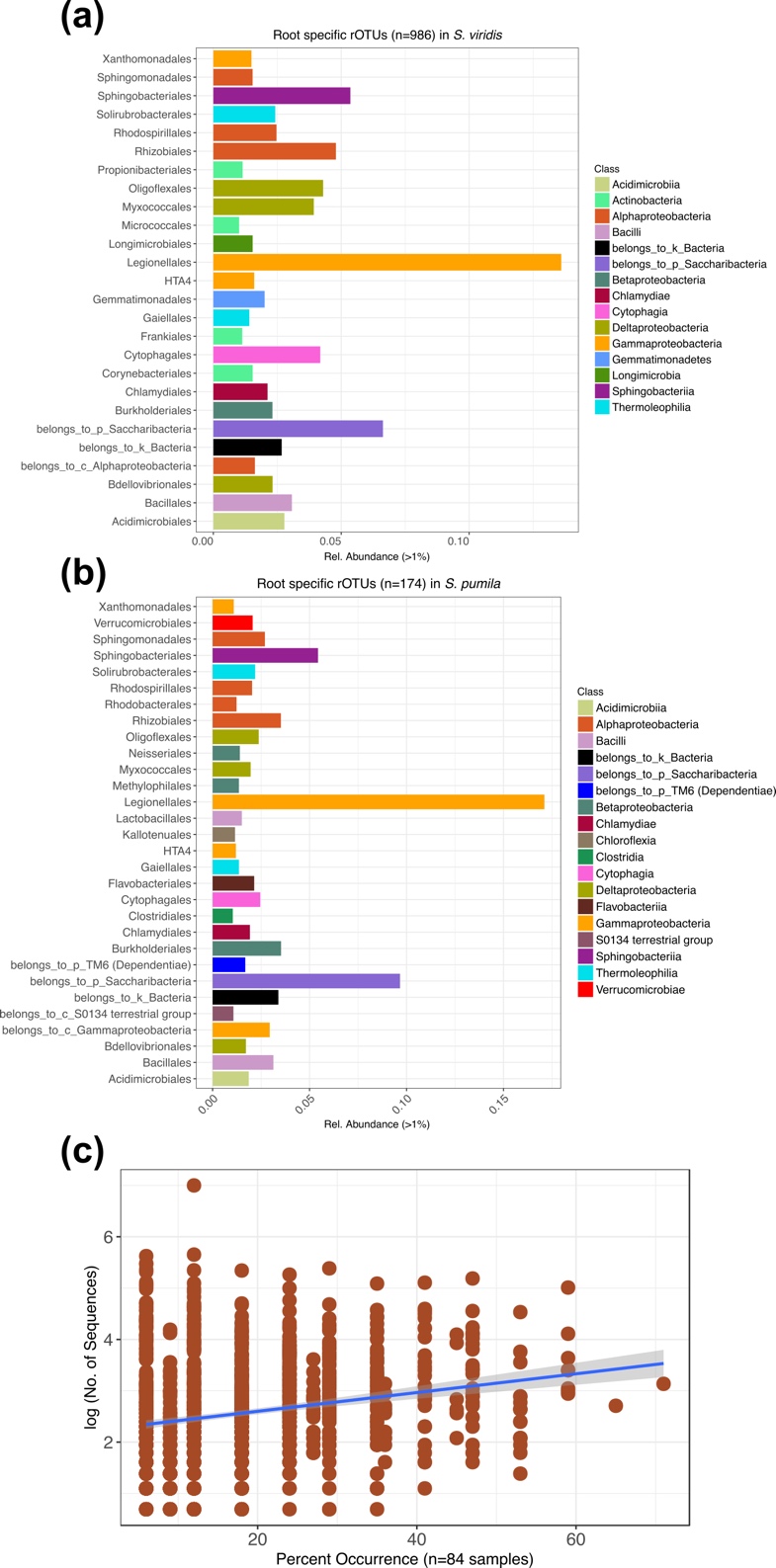


**Fig. S8.** Root-specific rOTU communities in *S. viridis* and *S. pumila*. **(a)** Relative abundances of bacterial orders of root-specific communities of *S. viridis***. (b)** Relative abundances of bacterial orders of root-specific communities of *S. pumila* **(c)** Root-specific rOTUs (n=1160) that were characteristic for each plant species shower relatively low occurrences across locations. Each rOTU is a point, and the line shows the log-linear model for the occurrence / abundance relationship. The grey shading represents the standard error.

**
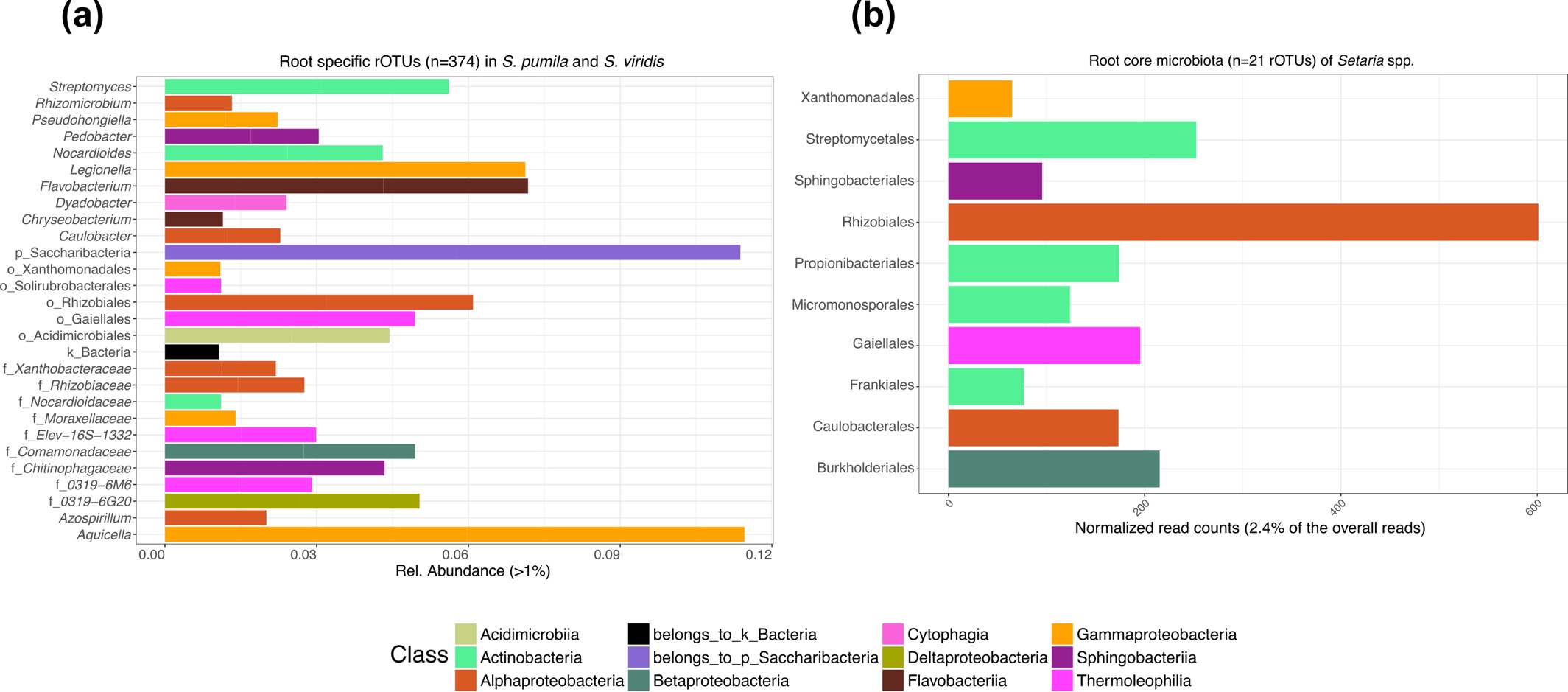
**

**Fig. S9.** Root-specific communities shared among samples of *S. pumila* and *S. viridis* **(a)** taxonomic distribution of root-specific communities based on genera **(b)** core-root rOTUs and their community structure based on bacterial orders*.* OTU counts were normalized by cumulative sum scaling (CSS).


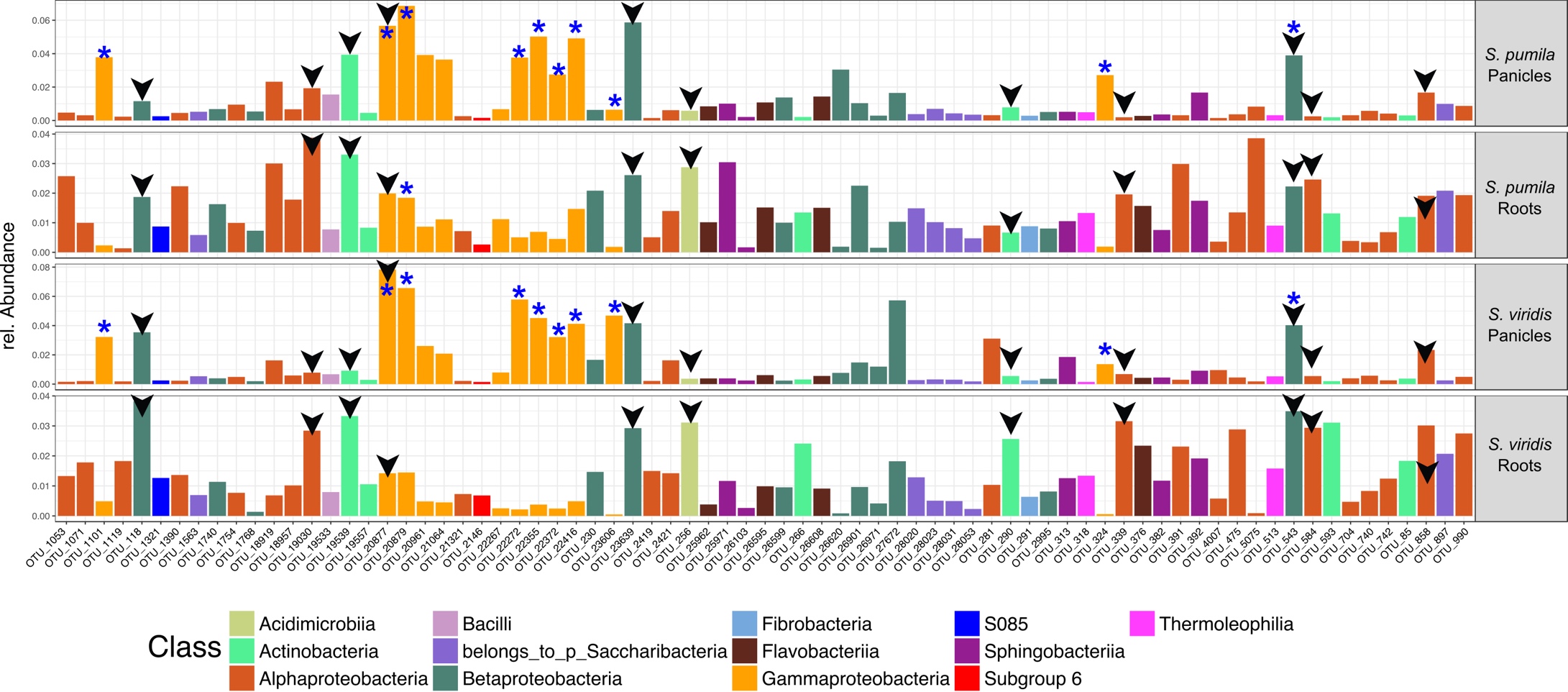


**Fig. S10.** Taxonomic classification and relative abundances of the 75 rOTUs present in all sample types. Black arrows point towards rOTUs present in samples of at least 80% of all locations. Blue stars highlight those rOTUs found to be significantly enriched in panicle samples.

**Table S6.** rOTUs which were significantly enriched in panicle tissues (among the 75 widey-distributed rOTUs). The complete list of differentially abundant rOTUs in panicles can be found in the supplementary file Data S2.

| **rOTU** | **Assigned taxonomy** | **Occurrence percentage across sampling locations** |
| --- | --- | --- |
| OTU_1101 | *Pseudomonas* | **60%** |
| OTU_20877 | ***Psychrobacter*** | **85%** |
| OTU_20879 | *Halomonas* | **75%** |
| OTU_22272 | f_*Enterobacteriaceae* | **75%** |
| OTU_22355 | f_*Enterobacteriaceae* | **65%** |
| OTU_22372 | f_*Enterobacteriaceae* | **55%** |
| OTU_22416 | *Pantoea* | **65%** |
| OTU_23606 | f_*Enterobacteriaceae* | **50%** |
| OTU_324 | *Pseudomonas* | **50%** |
| OTU_543 | ***Massilia*** | **90%** |
